# Supplementary material for: Coordination environment dependent selectivity of single-site-Cu enriched crystalline porous catalysts in CO2 reduction to CH4
Source: Nat Commun. 2021 Nov 4;12:6390. doi: 10.1038/s41467-021-26724-8 (PMC8568903; doi:10.1038/s41467-021-26724-8)
Supplement: Supplementary file 1 — Supplementary Information [file 41467_2021_26724_MOESM1_ESM.pdf]

1. Supplementary Information

1.1 Supplementary Methods Details

Materials

Synthesis methods

Electrochemical measurements

Standard curves on GC

Computational methods

1.2 Supplementary Figures and Tables

Supplementary Fig. 1 The topological structure of Cu-DBC.

Supplementary Fig. 2 The N_2 adsorption-desorption isotherm and pore size distribution of Cu-DBC catalyst.

Supplementary Fig. 3 The CO_2 adsorption-desorption isotherm of Cu-DBC catalyst.

Supplementary Fig. 4 SEM image of Cu-DBC.

Supplementary Fig. 5 The corresponding EDS element mapping in Fig. 1e.

Supplementary Fig. 6 XPS analysis.

Supplementary Table 1 ICP-AES test results of Cu-DBC catalyst.

Supplementary Fig. 7 TGA curve of Cu-DBC tested under Ar.

Supplementary Fig. 8 Schematic illustration of electrochemical setup.

Supplementary Fig. 9 Chronoamperometric curves at various potentials for ECR over Cu-DBC electrocatalyst.

Supplementary Fig. 10 ^{1}H NMR spectrum of the filtrate after ECR stability test at -0.9 V vs. RHE over Cu-DBC electrocatalyst.

Supplementary Fig. 11 Mass spectra extracted from GC-MS analysis of products from $^{13}CO_2$ reduction.

Supplementary Fig. 12 SEM images of the Cu-DBC electrode

Supplementary Fig. 13 Raman analysis.

Supplementary Fig. 14 XPS analysis.

Supplementary Table 2 Cu 2*p* XPS spectra peak fit parameters of Cu-DBC before and after electrocatalysis

Supplementary Fig. 15 Characterizations of the as-prepared Cu-HHTP.

Supplementary Fig. 16 Characterizations of the as-prepared Cu-TTCOF.

Supplementary Fig. 17 Characterizations of the as-prepared Cu-PPCOF.

Supplementary Fig. 18 ECR Electrochemical performance of Cu-HHTP.

Supplementary Fig. 19 EIS curves tested over the Cu-DBC and Cu-HHTP catalysts at a potential of -0.9 V vs. RHE.

Supplementary Fig. 20 ECR Electrochemical performance of Cu-PPCOF.

Supplementary Fig. 21 ECR Electrochemical performance of Cu-TTCOF.

Supplementary Fig. 22 Electrochemically active surface area (ECSA) analysis of Cu-DBC.

Supplementary Fig. 23 Electrochemically active surface area (ECSA) analysis of Cu-HHTP.

Supplementary Fig. 24 Electrochemically active surface area (ECSA) analysis of Cu-TTCOF.

Supplementary Fig. 25 Electrochemically active surface area (ECSA) analysis of Cu-PPCOF.

Supplementary Fig. 26 XRD patterns of fresh and tested catalysts modified GDL-carbon paper electrodes.

Supplementary Fig. 27 SEM images.

Supplementary Fig. 28 CV curves of Cu-DBC under the voltage window with and without ECR region.

Supplementary Fig. 29 Redox properties analysis of Cu-DBC.

Supplementary Fig. 30 Modeled structure (left) and optimized structure (right) for *H adsorption on catalytic sites.

Supplementary Table 3 Löwdin charge analysis of the Cu-O_4 and Cu-N_4 systems.

Supplementary Fig. 31 The energy barrier of the formation of H_2 (i.e. full HER process) for Cu-O_4 system following the Volmer-Tafel mechanism.

Supplementary Fig. 32 The ananlysis of electroreduction active sites in Cu-PPCOF.

Supplementary Fig. 33 Structures of the reaction intermediates involved in the proposed reaction mechanism for the ECR-to-CH_4 on Cu-O_4 site in Cu-DBC catalyst.

Supplementary Fig. 34 Structures of the reaction intermediates involved in the proposed reaction mechanism for the ECR-to-CH_4 on Cu-N_4 site in Cu-TTCOF catalyst.

Supplementary Fig. 35 The energy profiles of Cu-O_4 sites in Cu-DBC with $U = 0$ and $U = U_{Onset}$.

Supplementary Fig. 36 The energy profiles of porphyrin Cu-N_4 in Cu-TTCOF with $U = 0$ and $U = U_{Onset}$.

Supplementary Fig. 37 The energy profiles of phthalocyanine Cu-N_4 in Cu-PPCOF with $U = 0$ and $U = U_{Onset}$.

Supplementary Table 4 Electrochemical performance of CO_2 reduction to CH_4 of Cu-based electrocatalysts

1.3 The structure models (with coordinates) of DFT calculations.

2. Supplementary Movie 1 The stretching vibrations of Peak 1 in Supplementary Fig. 13b.

3. Supplementary Movie 2 The stretching vibrations of Peak 2 in Supplementary Fig. 13b.

4. Supplementary Movie 3 The stretching vibrations of Peak 3 in Supplementary Fig. 13b.

Supplementary Information

Coordination Environment Dependent Selectivity of Single-Site-Cu Enriched Crystalline Porous Catalysts in CO_2 Reduction to CH_4

Yu Zhang[1], Long-Zhang Dong[1], Shan Li[1], Xin Huang[1], Jia-Nan Chang[1], Jian-Hui Wang[1], Jie Zhou[2], Shun-Li Li[1] & Ya-Qian Lan[1,2*]

[1]Jiangsu Collaborative Innovation Centre of Biomedical Functional Materials, Jiangsu Key Laboratory of New Power Batteries, School of Chemistry and Materials Science, Nanjing Normal University, Nanjing 210023, P. R. China.

[2]School of Chemistry, South China Normal University, Guangzhou, 510006, P. R. China.

Yu Zhang and Long-Zhang Dong contributed equally to this work. Correspondence and requests for materials should be addressed to Y.-Q. L. (Email: yqlan@njnu.edu.cn).

Supplementary Methods Details

Materials

All solvents and reagents obtained from commercial sources were used without further purification. Dibenzo-[g,p]chrysene-2,3,6,7,10,11,14,15-octaol (8OH-DBC) was purchased from Jinlin Chinese Academy of Science-Yanshen Technology Co., Ltd. 2,3,6,7,10,11-hexahydroxytriphenylene (HHTP) and 2,3,6,7,10,11-hexaaminotriphenylene hexahydrochloride (HATP·6HCl) were purchased from Kaiyulin (Shanghai) Development Co., Ltd. Cupric acetate monohydrate ($Cu(OAc)_2 \cdot H_2O$), copper(II) trifluoroacetylacetonate ($Cu(C_5H_4F_3O_2)_2$), 2,5-dihydroxyterephthalic acid (H_2dhtp), N,N-dimethylformamide (DMF, 99.5%), flake graphite, potassium hydroxide (KOH) and Na_2SO_4 were purchased from China National Medicines Corporation LTD. Nafion (5% w/w in water and 1-propanol) was purchased from Alfa Aesar Chemical Corporation LTD.

Synthesis methods

Synthesis of Cu-DBC. The synthesis of Cu-DBC was reported.[1] Typically, 8.6 mg of 8OH-DBC and 6 mg of $Cu(OAc)_2 \cdot H_2O$ were dispersed in 500 μL degassed dimethyformamide (DMF) and 2 mL degassed deionized water by ultrasonic treatment for 30 min. This vial was placed in 85 °C oven for 72 h. The reactant was washed with water and acetone several times, and dried overnight in vacuum at room temperature to obtain the black product.

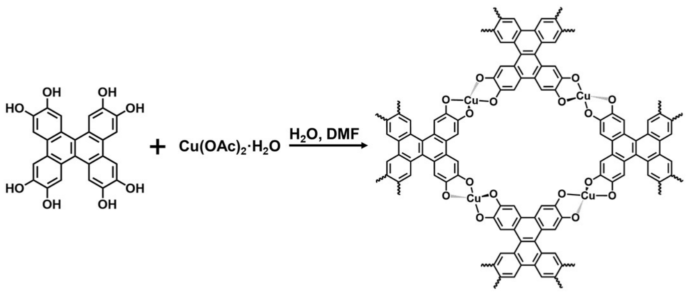

Synthesis of Cu-HHTP. The synthesis of Cu-HHTP was reported.[2] Typically, A solid mixture of HHTP (7.5 mg) and $Cu(C_5H_4F_3O_2)_2$ (10.5 mg) was dissolved in 1 mL of deionized water in a 4-mL glass vial. To this solution, 0.10 mL of NMP was added dropwise. The vial was then vigorously swirled and shortly sonicated resulting in a dark

solution. The reaction mixture was heated in an isothermal oven at 85 °C for 12 h resulting in dark blue crystals. The reaction mixture was allowed to cool naturally to room temperature and the crystals were washed with deionized water, and then, acetone and dried in air.

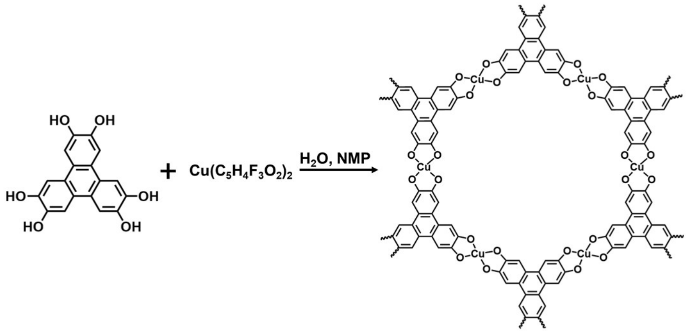

Synthesis of Cu-TTCOF. The synthesis of Cu-TTCOF followed our previously reported method.[3] A Pyrex tube measuring 19 × 65 mm (o.d × length) was charged with Cu-TAPP (14.8 mg, 0.02 mmol), TTF-4CHO (12.4 mg, 0.02 mmol), 1,4-dioxane (0.5 mL), 1,3,5-trimethylbenzene (0.5 mL) with ultrasonic for ~5 min to dissolve, then 6 M aqueous acetic acid (0.2 mL) were added. After sonication for another 15 minutes, the tube was flash frozen at 77 K (liquid N_2 bath) and degassed by three freeze-pump-thaw cycles and evacuated to an internal pressure of ~100 mTorr and sealed. After warmed to room temperature, the mixture was heated at 120 °C and left undisturbed for 72 hours. The obtained precipitate was isolated by filtration in Buchner funnel and was washed with THF and acetone until the filtrate was colorless. The wet sample was transferred to a Soxhlet extractor and washed with THF (24 hours) and acetone (24 hours). Finally, the product was evacuated at 150 °C under dynamic vacuum overnight to yield activated sample.

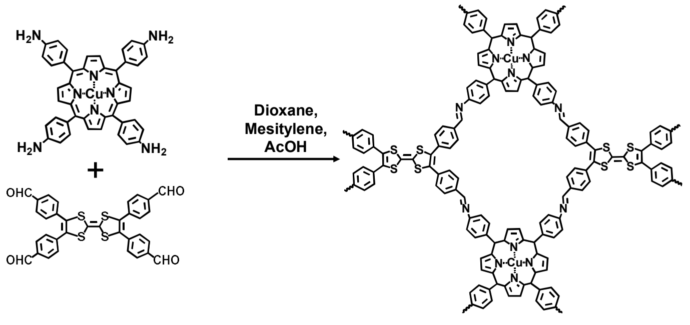

Synthesis of Cu-PPCOF. The synthesis of Cu-PPCOF was conducted via a modified

method according to the previous reported.[4] Typically, polycondensation of copper(II) 2,3,9,10,16,17,23,24-octakis(amino) phthalocyanine ($[NH_2]_8CuPc$) and 4,5,9,10-pyrenediquinone (PDQ) were added in the mixture of dimethylacetamide (DMAc) and ethylene glycol with acetic acid catalyst at 200°C. After one week, Cu-PPCOF was collected as grey-green powder in a yield of 70%. The wet sample was transferred to a Soxhlet extractor and washed with THF (24 hours) and acetone (24 hours). Finally, the product was evacuated at 80 °C under dynamic vacuum overnight to yield activated sample.

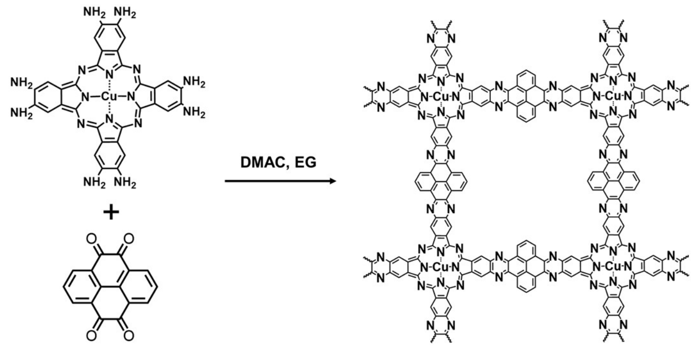

Electrochemical measurements

10 mg of electrocatalyst was grinded to powered and then dispersed into H_2O/ethanol (950 uL, V/V=1:1) followed by adding 50 μL Nafion, after mixing with assistance of ultrasonication for at least 30 min to achieve a homogeneous ink. 50 μL of the catalyst ink was drop onto a commercial gas-diffusion layer electrode (S = 0.25 cm × 1 cm) to form the work electrode.

CO_2 electroreduction was performed in a three-electrode flow cell. The work electrode area was 0.25 cm^2 and the distance from work electrode to the membrane was 1.5 mm. An external Ag/AgCl electrode (CHI111) located ≈3 cm from the cathode was acting as reference electrode. All potentials were converted to the reversible hydrogen electrode (RHE) in scale according to the following equation

$$E\ (vs\ \text{RHE}) = E\ (vs\ \text{Ag/AgCl}) + 0.197\ \text{V} + 0.0591\ \text{V} \times \text{pH}$$

During the electrocatalysis, 1 M KOH aqueous electrolyte pumped via a peristaltic pump (LongerPump, BT100-2J) with a constant rate of 7 mL min^{-1} flowed through the cathode and anode chambers separately. And the flowing CO_2 gas pass through the GDL

with a rate of 20 sccm monitored by a mass flow controller (AITOLY, MFC300). An anion exchange membrane (Fumasep, FAA-3-PK-130) was used to separate the cathode and anode. The faradaic efficiencies (FEs) were calculated using the following equation:

$$\text{FE }\% = \frac{nFxV}{j_{Tot}} \times 100$$

Where n = number of electrons transferred

F = Faraday's constant

x = mole fraction of product

V = total molar flow rate of gas

j_{Tot} = total current density

The Ag/AgCl electrode (saturated KCl) was used as the reference electrode, and the counter electrode was matched with a Pt plate (1 cm^{-2}). Linear sweep voltammetry (LSV) curves were performed to choose the appropriate potential range for the catalysts. The sweeping range was from 0 to -1.0 V (*vs.* RHE) at a scan rate of 5 mV s^{-1} in 1 M KOH solution with CO_2/Ar flowing. The test of electrocatalytic CO_2RR and LSV were presented with 90% iR compensation at ambient pressure and room temperature. Cyclic voltammograms (CV) were tested in 1 M KOH solution with various scan rates without iR compensation. Before the CV test, the electrolyte was bubbled with Ar for 30 min.

Standard curves on GC

Four concentrations of standard mixed gases (**1~4**) contained H_2, CO, CH_4, C_2H_2, C_2H_4, C_2H_6, C_3H_6 and C_3H_8 with CO_2 balance were used to calculated the standard curves for GC measurements. The concentration of the components (mol/mol) and the final standard curves are listed in **Supplementary Table 2~3** and **Supplementary Fig. 9**. The standard curves of H_2, CO, CH_4, C_2H_4, C_2H_6, C_2H_2, C_3H_6 and C_3H_8. H_2 and CO are detected by TCD and all the hydrocarbons are detected by FID. The gas chromatograms of FID and TCD Win **Supplementary Fig. 10** show that all the tested substance can be completely baseline separated.

Computational methods

In the present work, all the calculations including structure optimization, free energy calculation, and Raman properties were performed by using DFT (density functional

theory) method with fragment models. The structure models (with coordinates) of the three systems are shown as below. The calculations were performed using the ORCA package employing the resolution of identity approximation.[5] All the DFT calculations were performed using the Becke's three-parameter hybrid functional with gradient corrections provided by Lee, Yang, and Parr (B3LYP) functional. Basis sets of def2-SVP were used to optimize the structures and def2-TZVP [6,7] were adopted for Cu, C, N, O and H atoms in the complexes with decontracted auxiliary def2-TZVP/J Coulomb fitting basis sets to correct the energies.[8] The DFT grid was set to GRID4, and the convergence threshold TIGHT was employed for the self-consistent field (SCF) and the optimization procedure. D3 dispersion correction developed by Grimme is included for weak interactions.[9] Vibrational frequency calculations of optimized structures were performed at the same level of theory to ascertain the presence of a local minimum, confirming that there is no imaginary frequency observed for the considered systems, and was used to generate the zero-point energies (ZPE), the Gibbs free energies (G) and zero-point corrections.

The computational hydrogen electrode (CHE) model that proposed by Nørskov et al.[10] was applied to describe the Gibbs reaction free energy of reaction for CO_2RR elementary steps involving ($H^+ + e^-$) pair transfer. The calculation for each step can be defined as $\Delta G_n(U) = \Delta G_n(U{=}0) + neU$, where e is the elementary charge of an electron, n is the number of ($H^+ + e^-$) pairs transferred in CO_2RR and U is the electrode potential versus the reversible hydrogen electrode (RHE). Specifically, $\Delta G = \Delta E + \Delta E_{\mathrm{ZPE}} - T\Delta S + \int C_P \mathrm{d}T$ at U=0 V, where ΔE is the reaction energy difference between the product and reactant of the CO_2RR occurring on catalysts, which can be directly obtained from DFT computations; ΔE_{ZPE}, $T\Delta S$ and $\int Cp\mathrm{d}T$ are zero-point energy correction, entropy correction and enthalpic temperature correction at T=298.15 K respectively, which were calculated from the vibrational frequencies. The ΔE_{ZPE}, $T\Delta S$ and $\int Cp\mathrm{d}T$ for each reaction intermediates can be calculated by the following equations, respectively:

$$E_{ZPE} = \frac{1}{2}\sum\nolimits_i h v_i \qquad (1)$$

$$-TS = K_B T \sum_i \ln(1 - e^{-\frac{hv_i}{K_B T}}) - \sum_i hv_i(\frac{1}{e^{\frac{hv_i}{K_B T}} - 1}) \quad (2)$$

$$\int C_p dT = \sum_i hv_i(\frac{1}{e^{\frac{hv_i}{K_B T}} - 1}) \quad (3)$$

where h, v and K_B are Planck constant, vibrational frequencies and Boltzmann constant, respectively.

Supplementary Figures and Tables

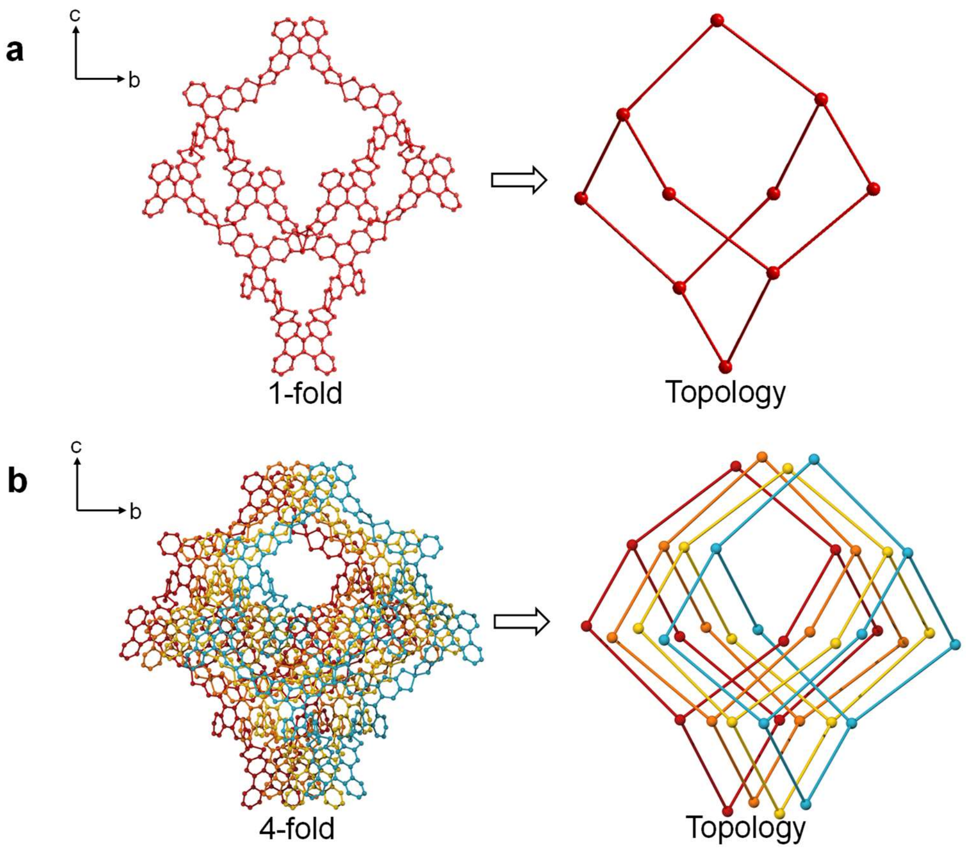

Supplementary Fig. 1 The topological structure of Cu-DBC. a The view of 1-fold structure. **b** the view of 4-fold interpenetrated structure.

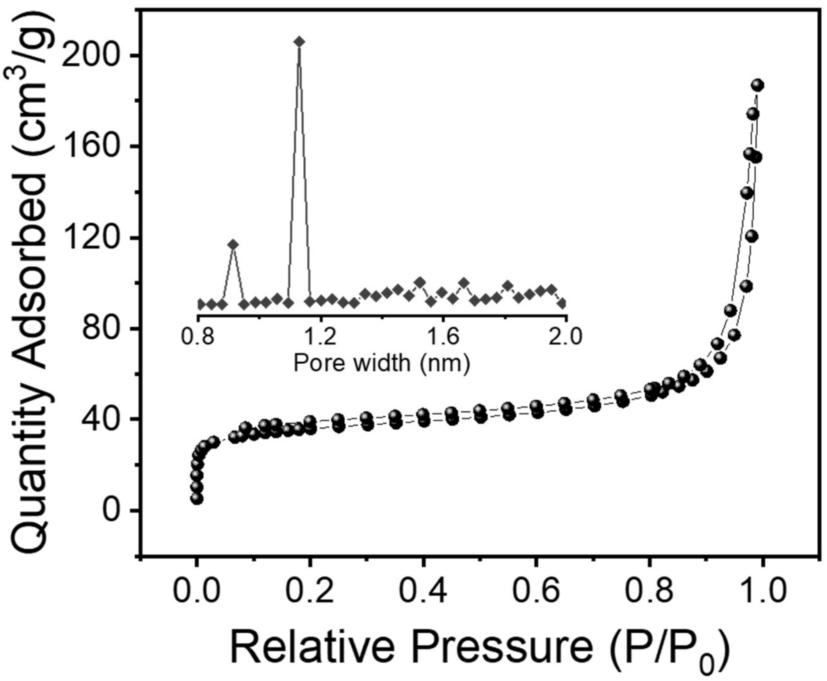

Supplementary Fig. 2 The N_2 adsorption-desorption isotherm and pore size distribution of Cu-DBC catalyst.

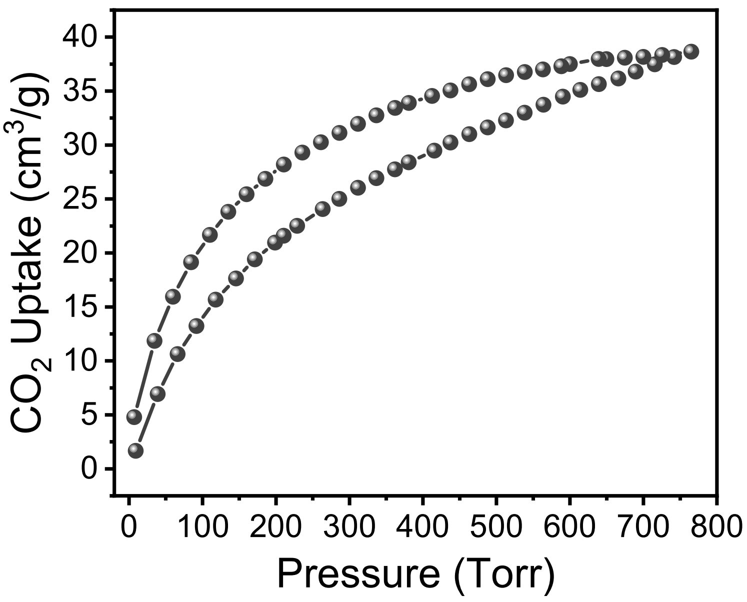

Supplementary Fig. 3 The CO_2 adsorption-desorption isotherm of Cu-DBC catalyst.

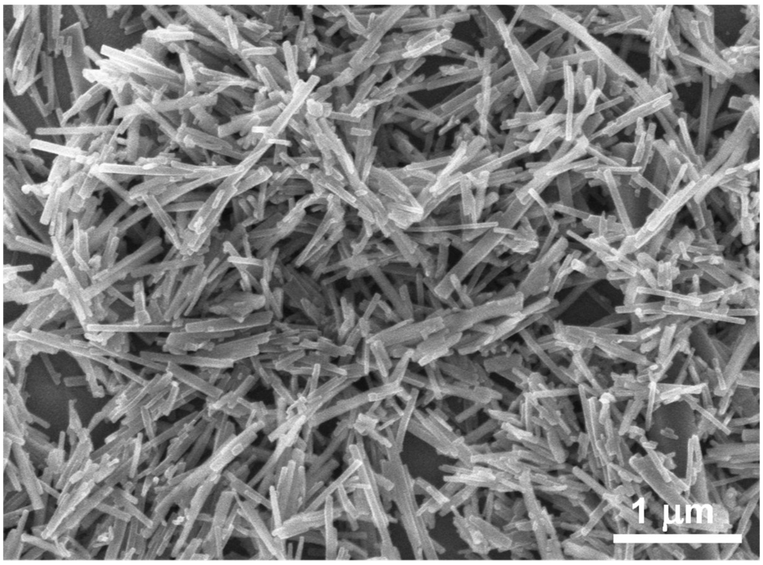

Supplementary Fig. 4 SEM image of Cu-DBC.

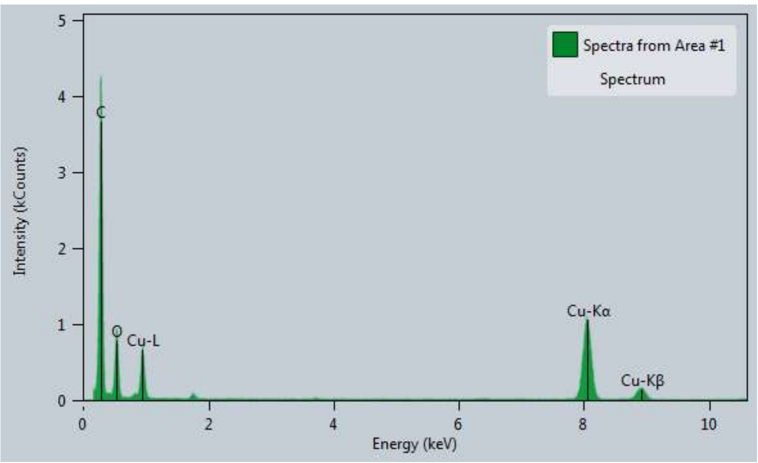

Supplementary Fig. 5 The corresponding EDS element mapping in Fig. 1e.

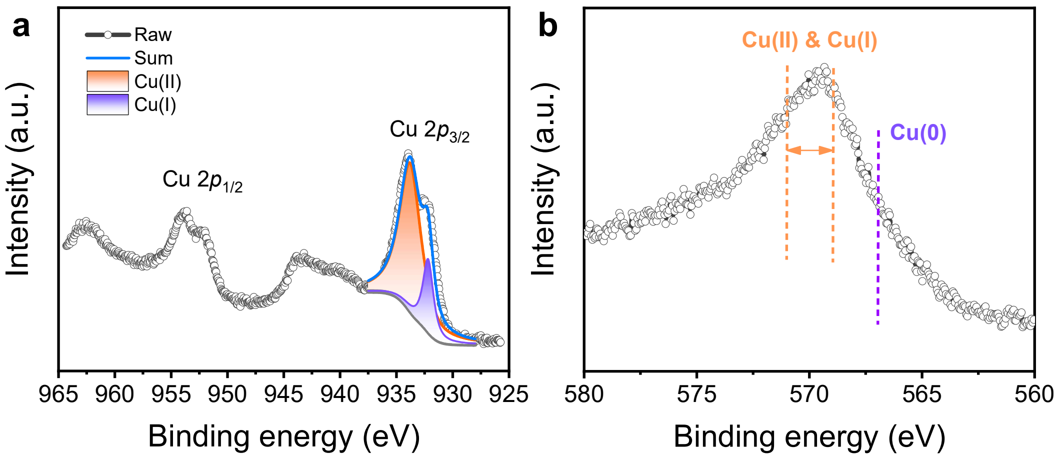

Supplementary Fig. 6 XPS analysis. a Cu 2*p* and **b** Auger Cu LMM XPS spectra of the Cu-DBC powder.

Inductively coupled plasma atomic emission spectrometry (ICP) was used to determine the content of metal element measured on an ICP Atomic Emission Spectrometer.

Supplementary Table 1 ICP-AES test results of Cu-DBC catalyst.

Sample	Cal. (wt.%)	Found (wt.%)
Cu-DBC	21.78	20.01

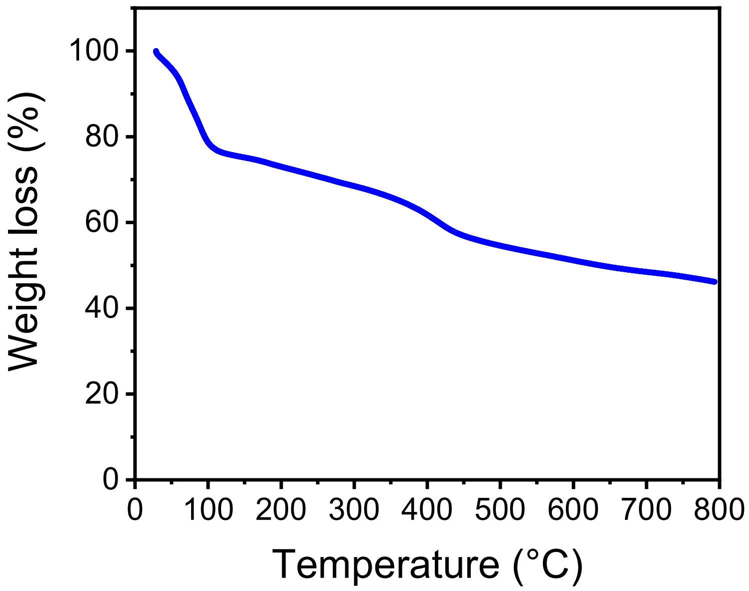

Supplementary Fig. 7 TGA curve of Cu-DBC tested under Ar.

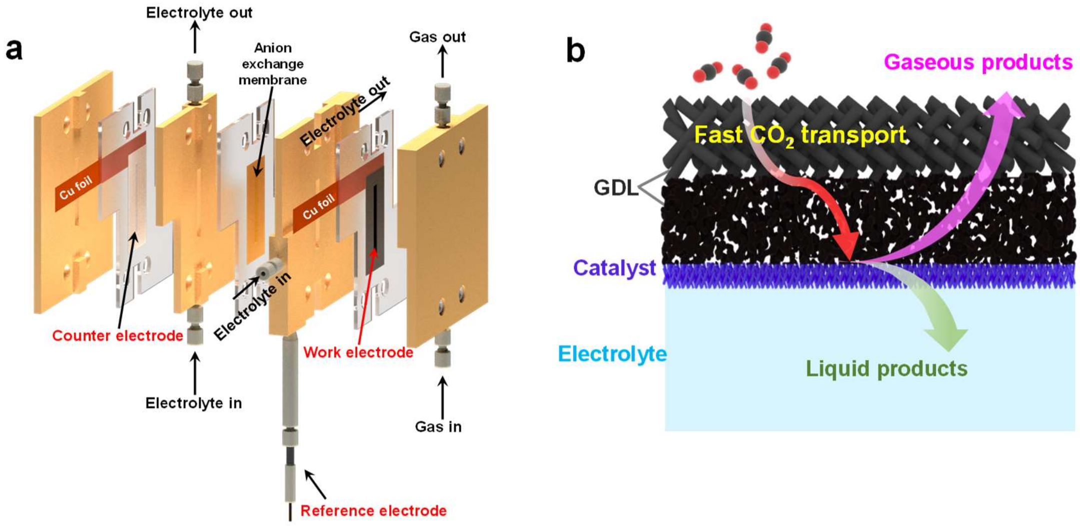

Supplementary Fig. 8 Schematic illustration of electrochemical setup. a flow cell setup and **b** gas diffusion layer.

The flow cell allows for the flowing gas (both CO_2 and products) passing through the gas diffusion layer of the work electrode in cathode chamber, which could avoid the pressure imbalance caused by gas products generation. Additionally, in contrast with H-type cell using saturated CO_2 bicarbonates electrolyte as CO_2 source, flow cell could provide enough amount CO_2 molecules by fast gas-phase transfer for the reaction and thus promote the mass transfer during catalysis process.

Supplementary Table 2 The data in standard curves of H_2, CO, CH_4 and C_2H_4.

	H_2	CO	CH_4	C_2H_4
**1**	9.94E-05	9.91E-05	1.01E-04	9.95E-05
**2**	1.01E-03	5.28E-04	5.29E-04	5.03E-04
**3**	9.82E-03	4.94E-03	5.06E-03	4.93E-03
**4**	4.98E-02	2.05E-02	1.99E-02	2.01E-02

Supplementary Table 3 The data in standard curves of C_2H_6, C_2H_2, C_3H_6 and C_3H_8.

	C_2H_6	C_2H_2	C_3H_6	C_3H_8
**1**	1.02E-04	9.99E-05	9.82E-05	9.92E-05
**2**	5.04E-04	5.15E-04	5.15E-04	5.15E-04
**3**	4.94E-03	4.91E-03	5.00E-03	4.88E-03
**4**	1.99E-02	1.95E-02	1.99E-02	1.98E-02

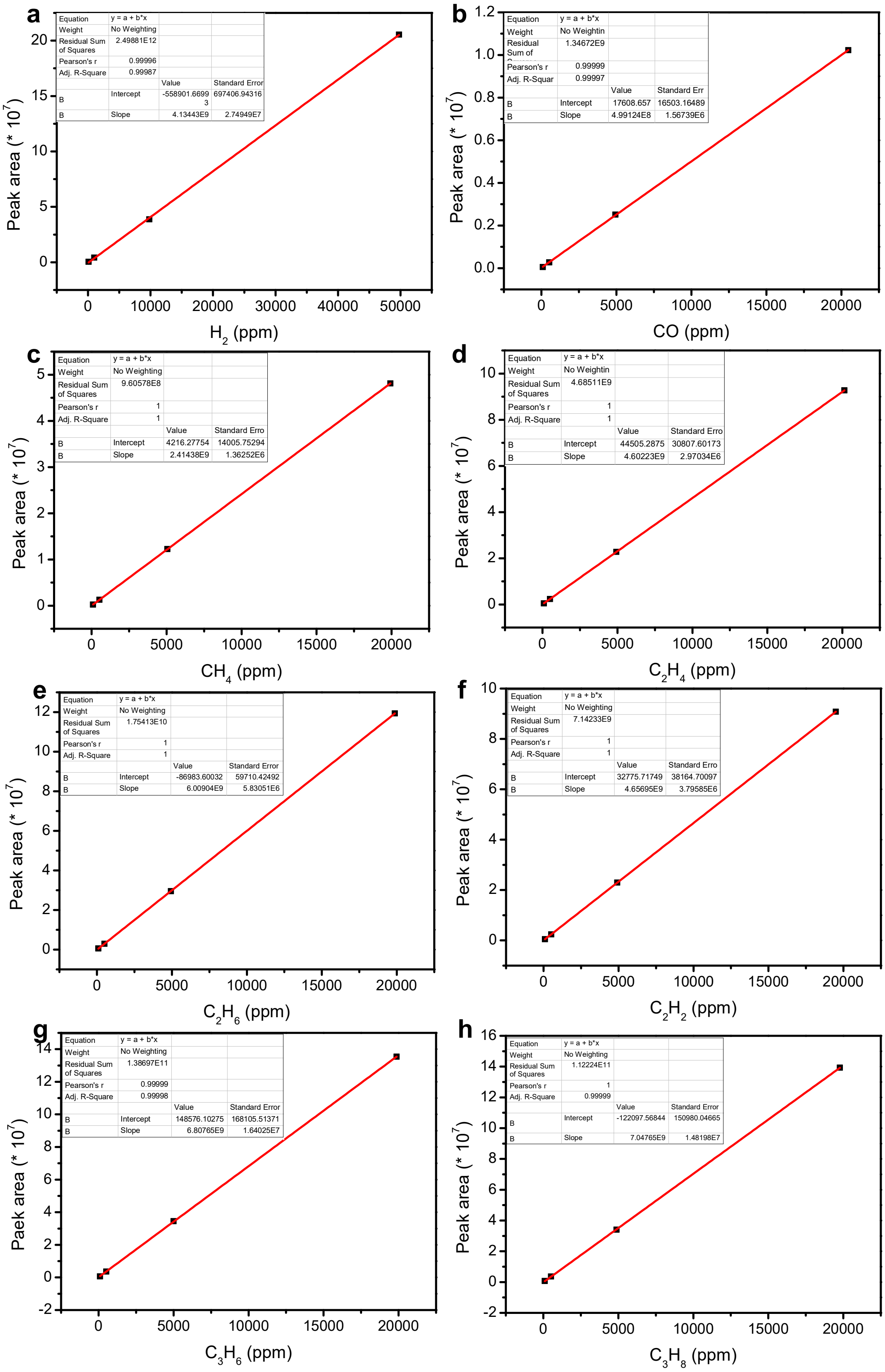

Supplementary Fig. 9 The standard curves on GC. a H_2, **b** CO, **c** CH_4, **d** C_2H_4, **e** C_2H_6, **f** C_2H_2, **g** C_3H_6 and **h** C_3H_8.

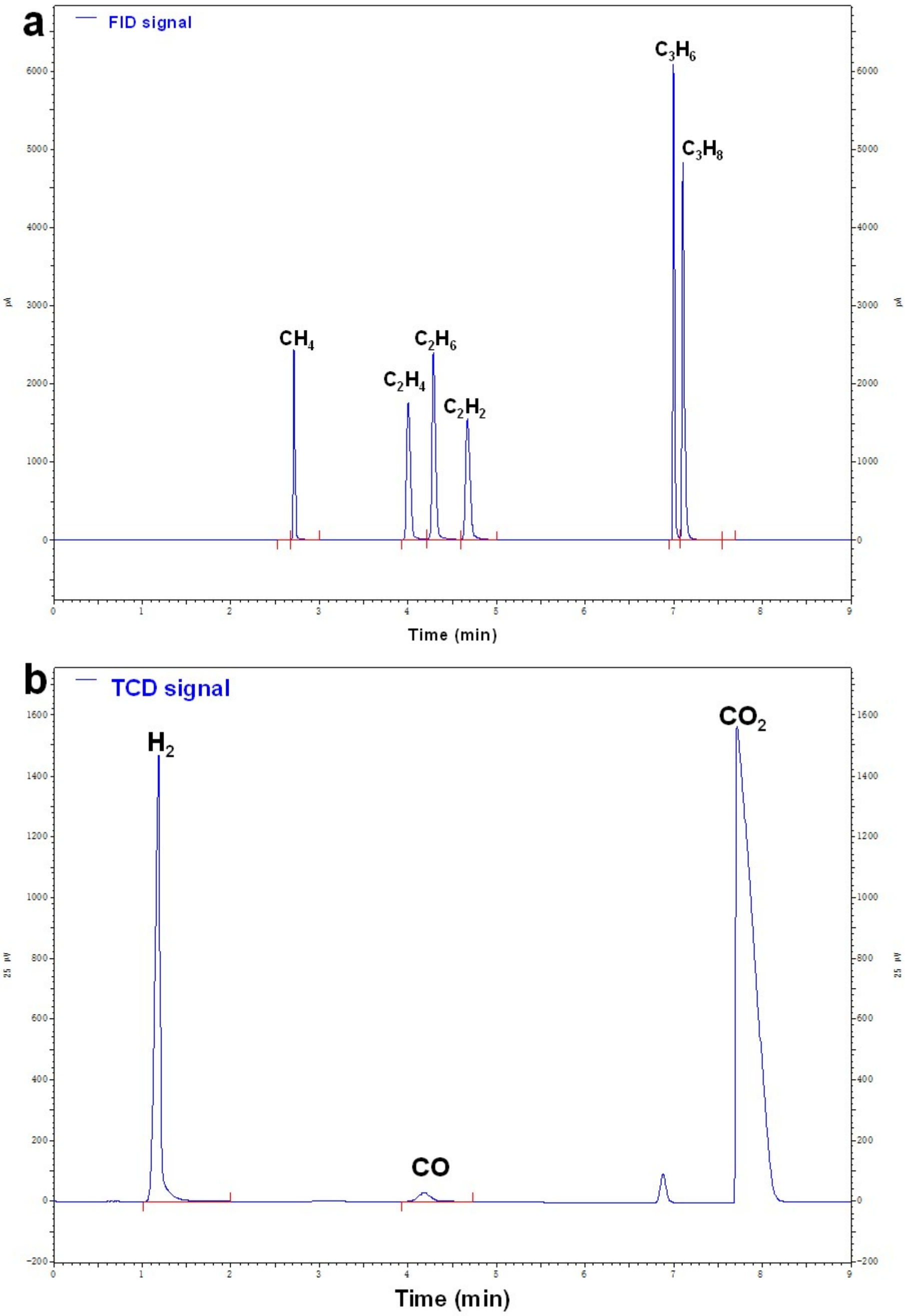

Supplementary Fig. 10 The gas chromatograms. a FID and **b** TCD.

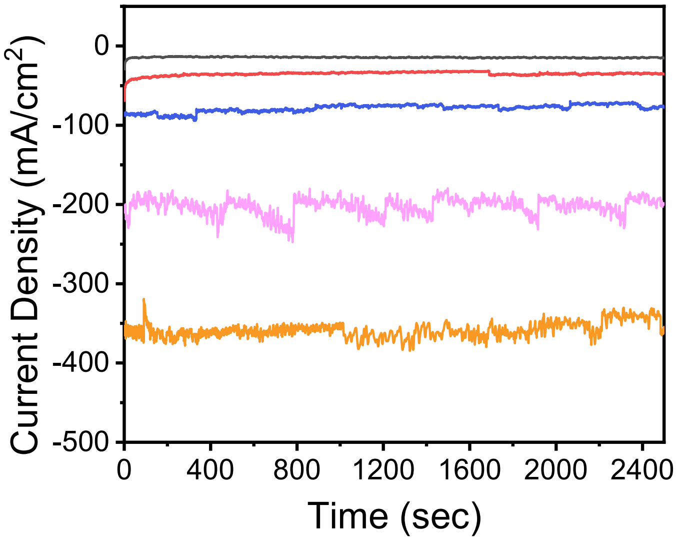

Supplementary Fig. 11 Chronoamperometric curves at various potentials for ECR over Cu-DBC electrocatalyst.

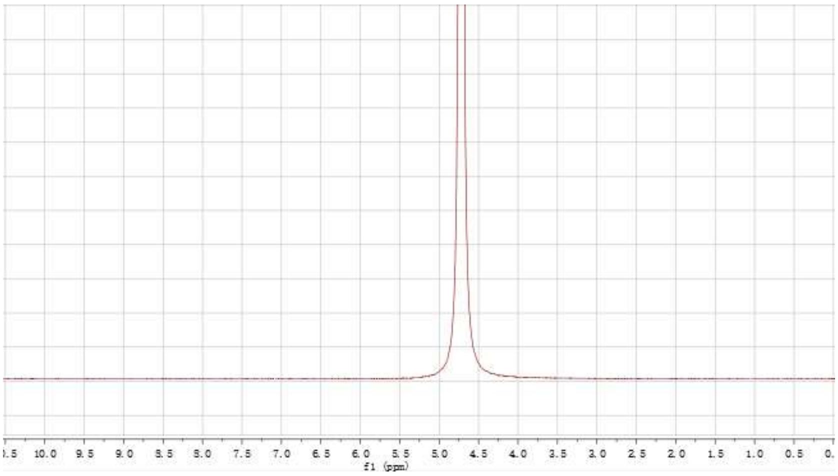

Supplementary Fig. 12 ^1H NMR spectrum of the filtrate after ECR stability test at -0.9 V vs. RHE over Cu-DBC electrocatalyst.

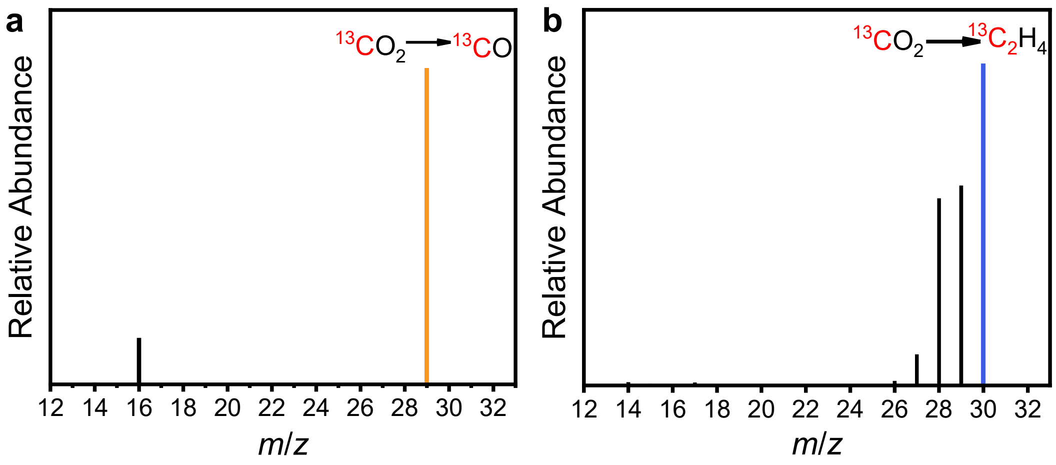

Supplementary Fig. 13 Mass spectra extracted from GC-MS analysis of products from $^{13}CO_2$ reduction. a CO and **b** C_2H_4.

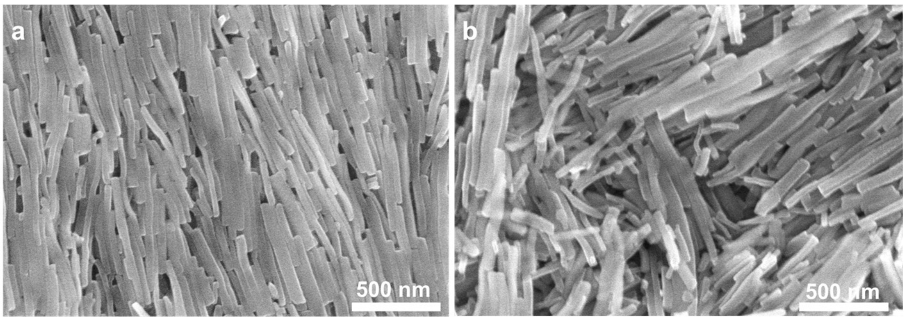

Supplementary Fig. 14 SEM images of the Cu-DBC electrode, a before and **b** after electrocatalysis.

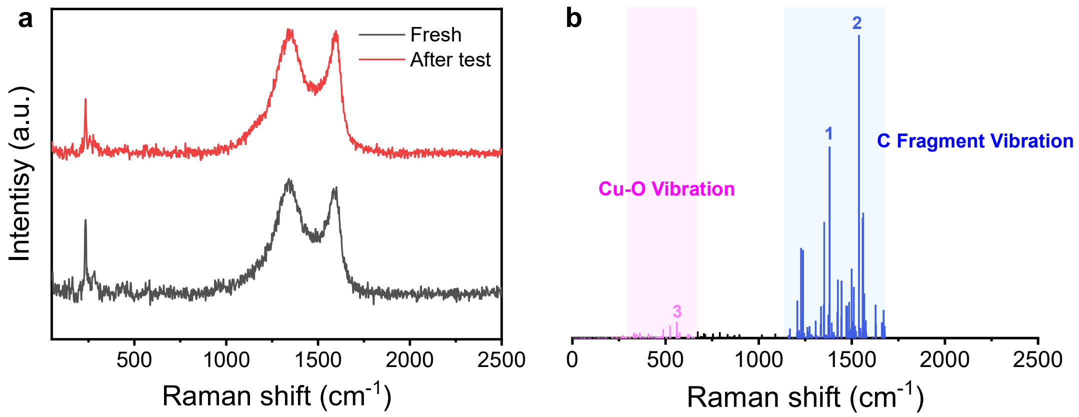

Supplementary Fig. 15 Raman analysis. a Raman spectra of Cu-DBC before and after electrocatalysis. **b** Simulated Raman spectrum based on Cu-O_4 site fragment in Cu-DBC system.

The calculation of theoretical Raman spectrum of Cu-DBC was conducted by DFT calculations. The fragment model extracted from crystal structures of the Cu-DBC system is adopted. The harmonic approximation is applied to obtain the vibrations of the system. All these factors can cause a reasonable quantitative mismatch between theoretical calculations and experiments. The DFT simulations can provide a qualitative understanding of the Raman spectrum of Cu-DBC. As shown in the **Supplementary Fig. 15b**, the simulated Raman spectrum by DFT confirms two main regions assigned to the Cu-O (pink region) and C fragment (blue region) stretching vibrations, respectively. The strong peaks in blue region around ~1380 (Peak **1**) and ~1540 cm^{-1} (Peak **2**) are mainly attributed to the D and G peaks in C fragment as shown in **Supplementary Movie 1** and **Supplementary Movie 2**. The strongest signal of ~560 cm^{-1} (Peak **3**) is caused by the stretching vibrations of Cu-O as shown in **Supplementary Movie 3**.

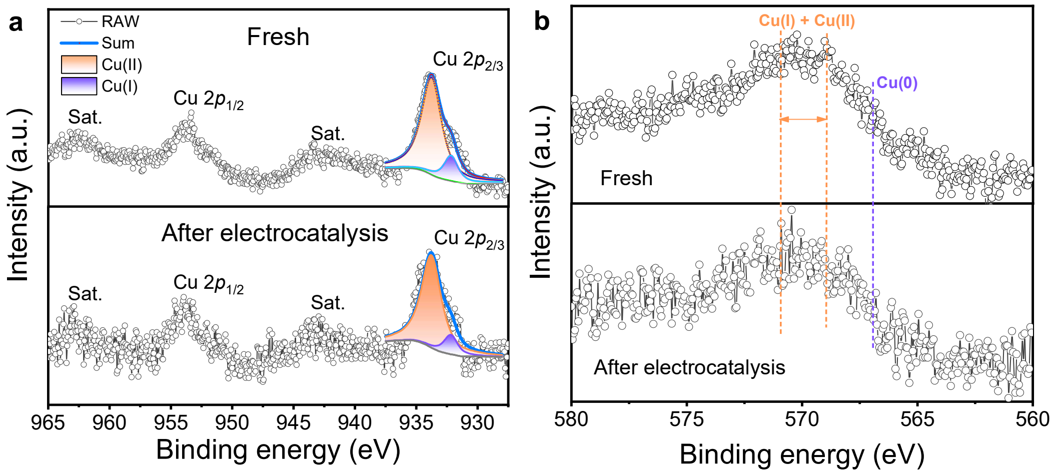

Supplementary Fig. 16 XPS analysis. a Cu 2*p* and **b** Auger Cu LMM XPS spectra of fresh and tested Cu-DBC modified GDL-CP electrodes.

Supplementary Table 4 Cu 2*p* XPS spectra peak fit parameters of Cu-DBC before and after electrocatalysis

Sample	Bind	Position	FWHM	Area (%)
Fresh	1-Cu(II)	933.77	1.92	84.6%
	2-Cu(I)	932.18	1.36	15.4%
After electrocatalysis	1-Cu(II)	933.77	1.92	86.9%
	2-Cu(I)	932.18	1.36	13.1%

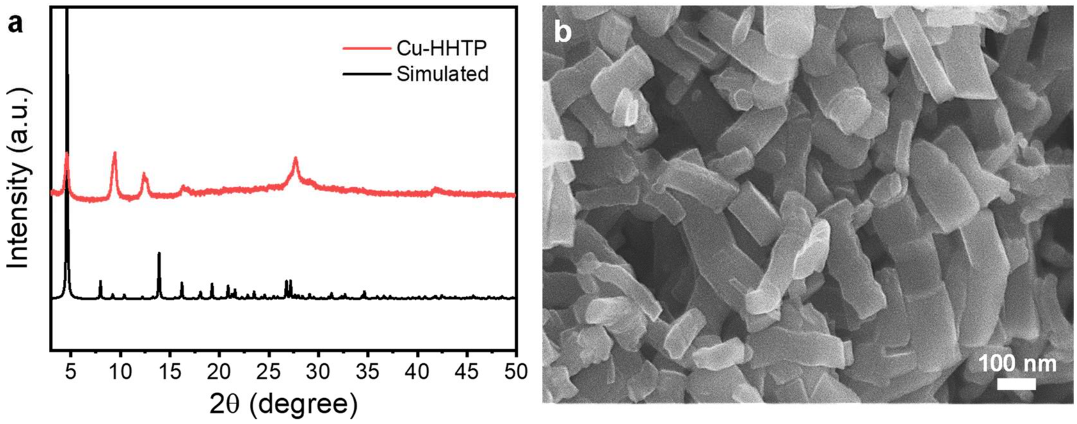

Supplementary Fig. 17 Characterizations of the as-prepared Cu-HHTP. a XRD patterns and **b** SEM image of Cu-HHTP.

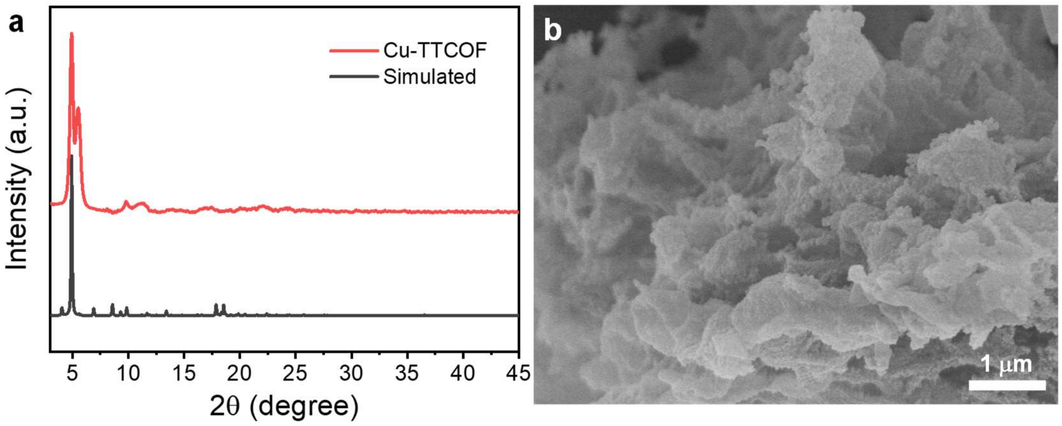

Supplementary Fig. 18 Characterizations of the as-prepared Cu-TTCOF. a XRD patterns and **b** SEM image.

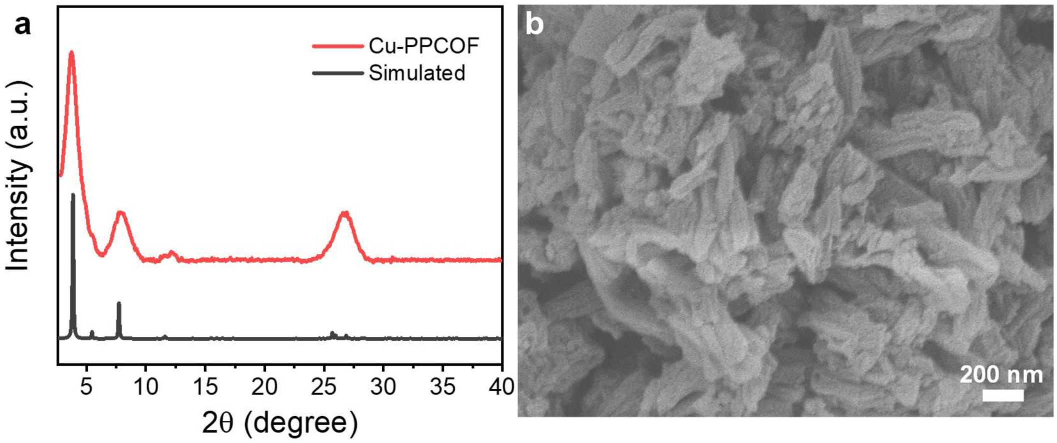

Supplementary Fig. 19 Characterizations of the as-prepared Cu-PPCOF. a XRD patterns and **b** SEM image.

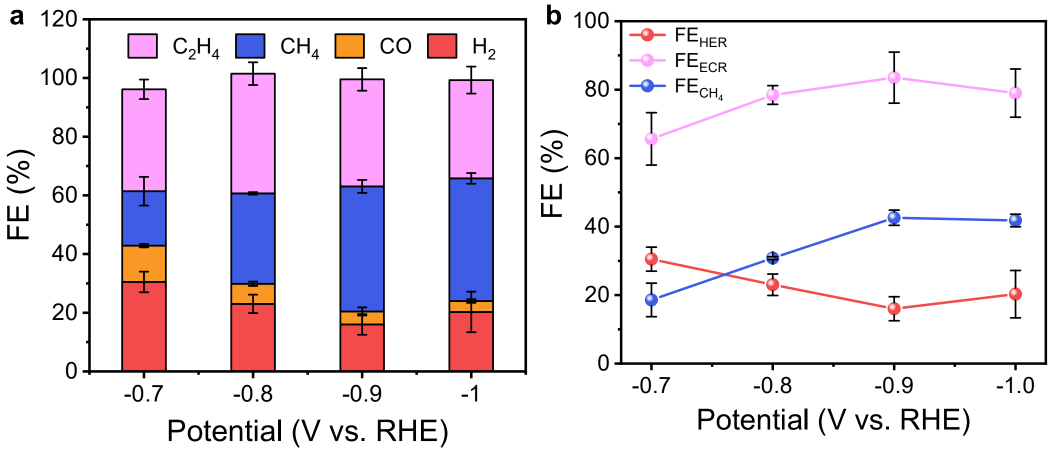

Supplementary Fig. 20 ECR Electrochemical performance of Cu-HHTP. a FEs of ECR products at different applied potentials. **b** FEs for HER, ECR and CH_4 recorded at different applied potentials.

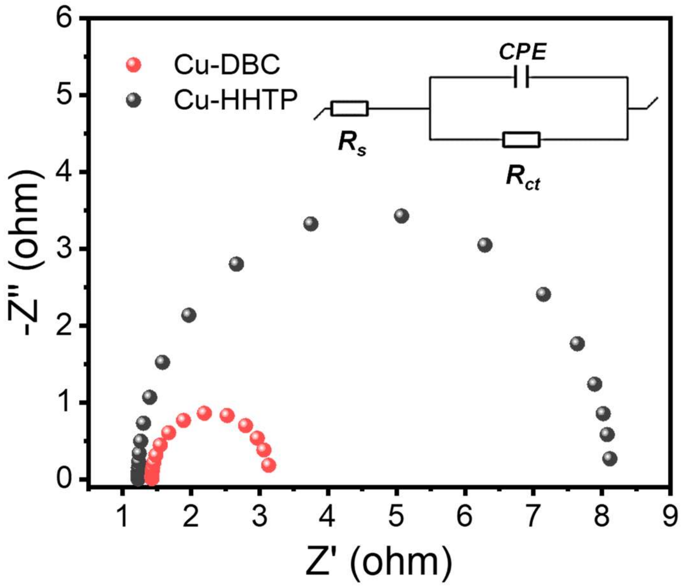

Supplementary Fig. 21 EIS curves tested over the Cu-DBC and Cu-HHTP catalysts at a potential of -0.9 V vs. RHE.

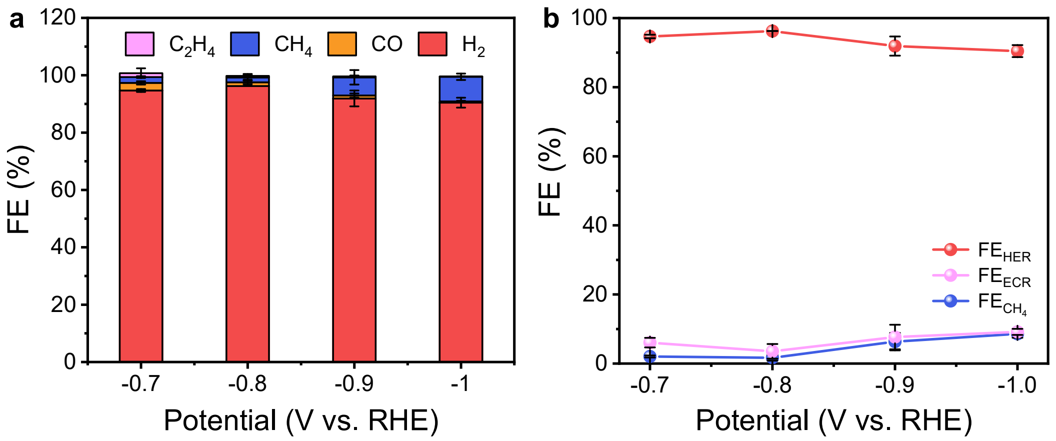

Supplementary Fig. 22 ECR Electrochemical performance of Cu-PPCOF. a FEs of ECR products at different applied potentials. **b** FEs for HER, ECR and CH_4 recorded at different applied potentials.

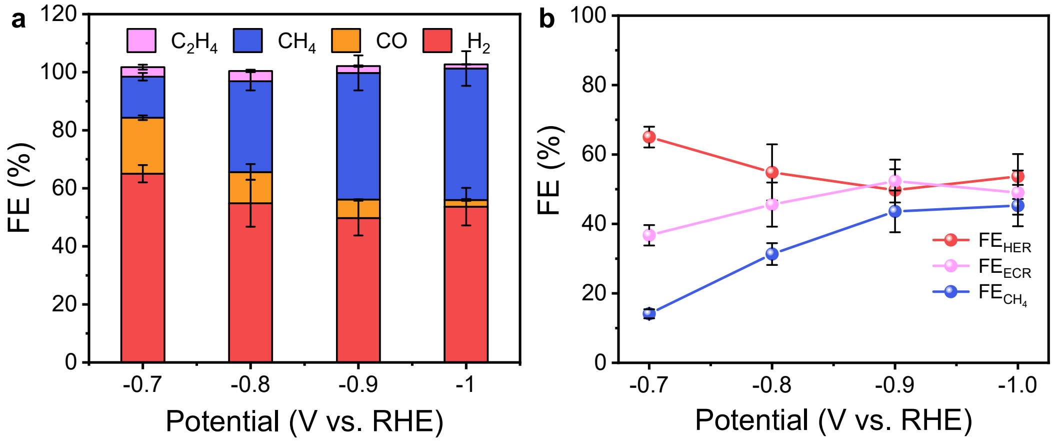

Supplementary Fig. 23 ECR Electrochemical performance of Cu-TTCOF. a FEs of ECR products at different applied potentials. **b** FEs for HER, ECR and CH_4 recorded at different applied potentials.

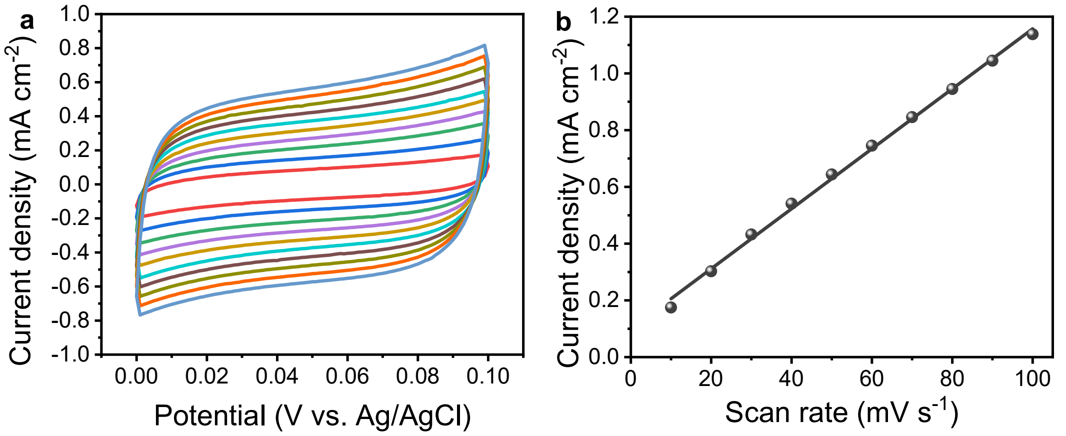

Supplementary Fig. 24 Electrochemically active surface area (ECSA) analysis of Cu-DBC. a Cyclic voltammertrys (CV) curves at various scan rate (10 ~ 100 mV s^{-1}). **b** The corresponding relationship of current density and scan rate.

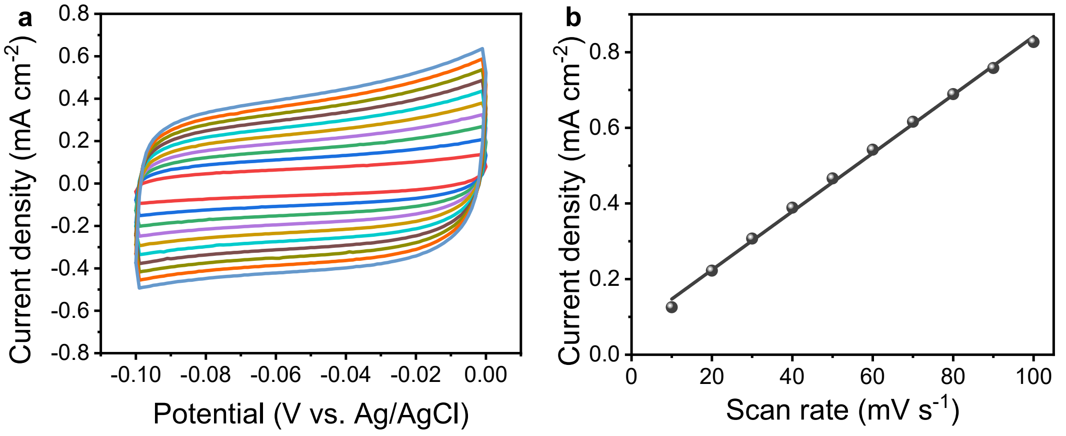

Supplementary Fig. 25 Electrochemically active surface area (ECSA) analysis of Cu-HHTP. a Cyclic voltammertrys (CV) curves at various scan rate (10 ~ 100 mV s^{-1}). **b** The corresponding relationship of current density and scan rate.

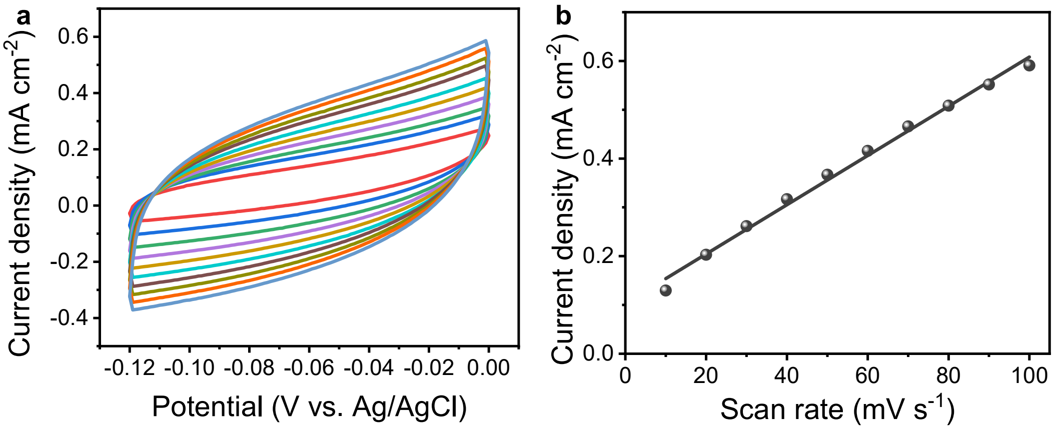

Supplementary Fig. 26 Electrochemically active surface area (ECSA) analysis of Cu-TTCOF. a Cyclic voltammertrys (CV) curves at various scan rate (10 ~ 100 mV s^{-1}). **b** The corresponding relationship of current density and scan rate.

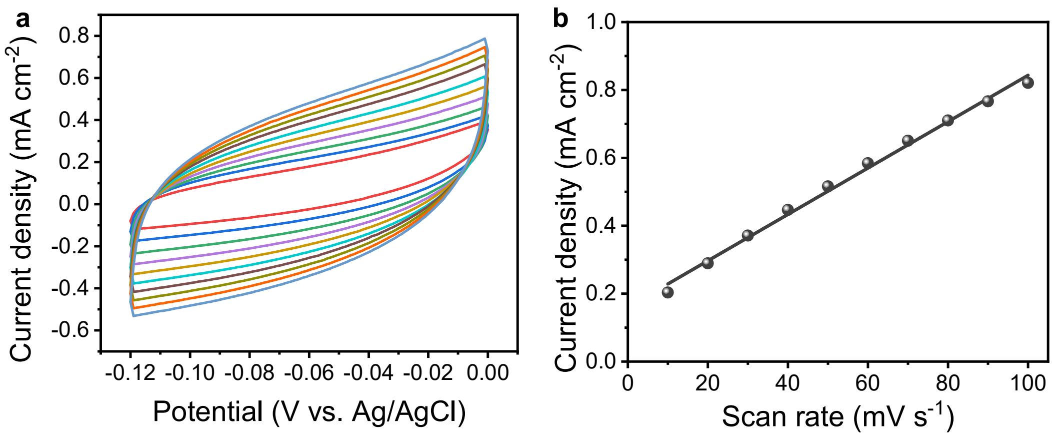

Supplementary Fig. 27 Electrochemically active surface area (ECSA) analysis of Cu-PPCOF. a Cyclic voltammertrys (CV) curves at various scan rate (10 ~ 100 mV s^{-1}). **b** The corresponding relationship of current density and scan rate.

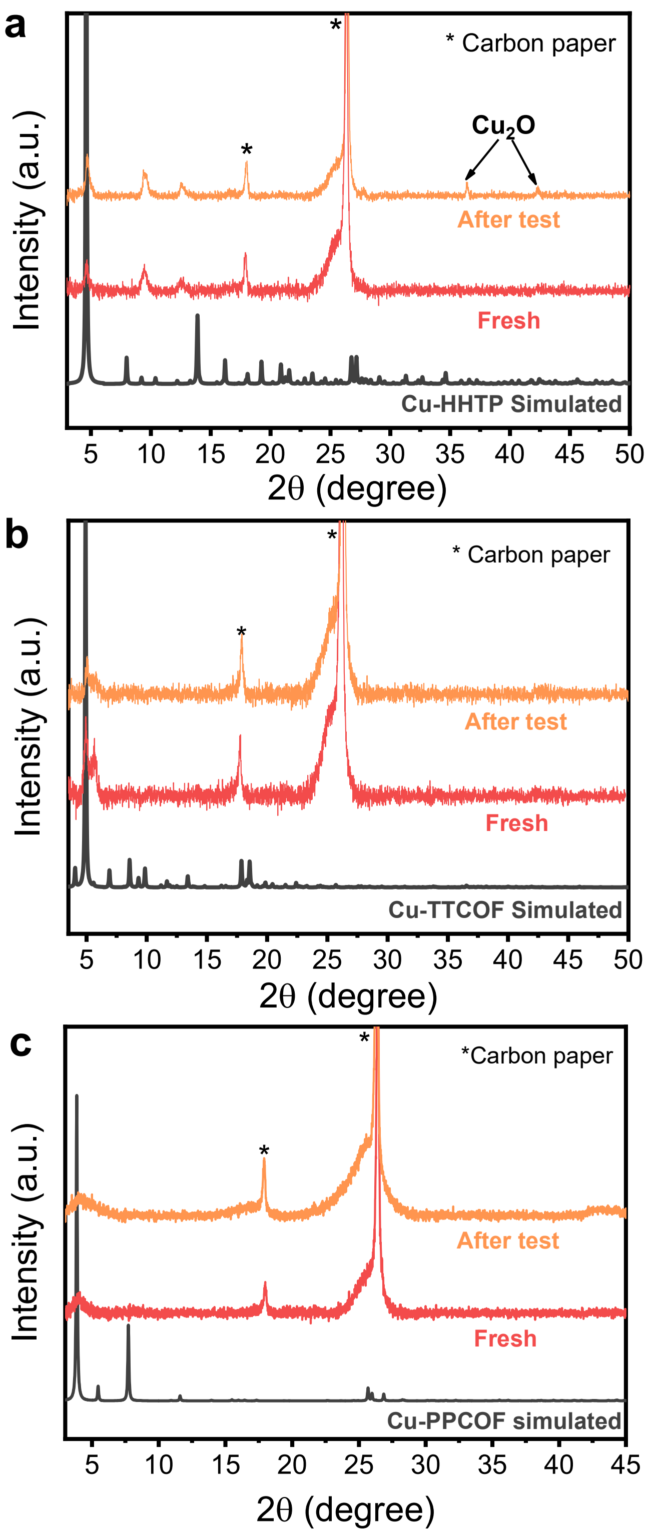

Supplementary Fig. 28 XRD patterns of fresh and tested catalysts modified GDL-carbon paper electrodes. a Cu-HHTP, **b** Cu-TTCOF, and **c** Cu-PPCOF

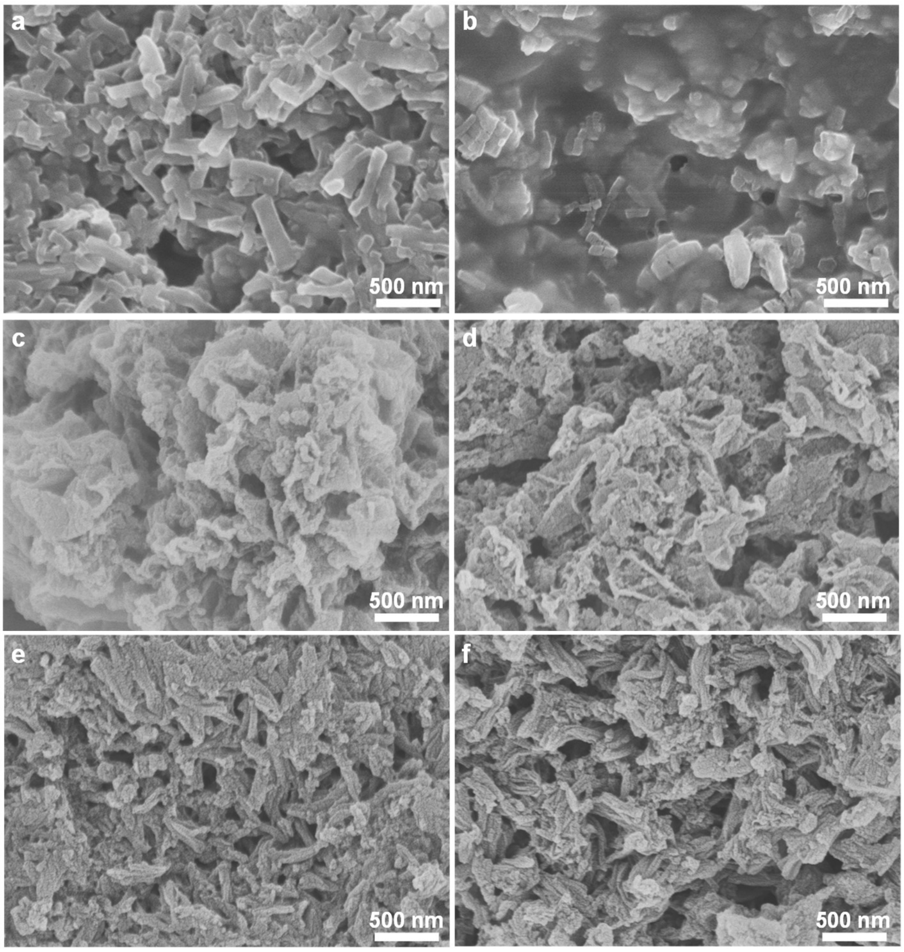

Supplementary Fig. 29 SEM images. a, b Cu-HHTP, **c, d** Cu-TTCOF, and **e, f** Cu-PPCOF electrocatalysts **a, c, e** before and **b, d, f** after electrocatalysis.

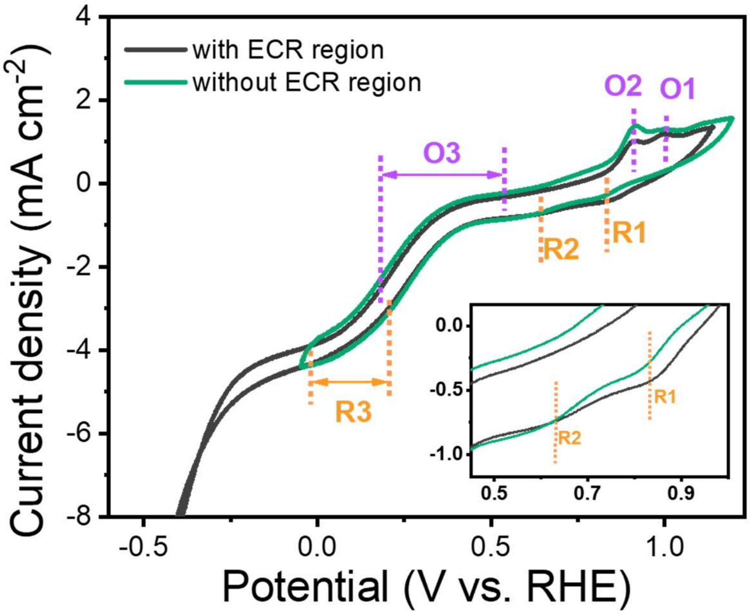

Supplementary Fig. 30 CV curves of Cu-DBC under the voltage window with and without ECR region.

The CV curves tested with and without ECR region over Cu-DBC electrocatalyst show similar redox peaks, indicating that the Cu-DBC exhibts reversible redox properties no matter it occurs ECR or not. It means that there is no other new Cu redox species generated during the ECR process.

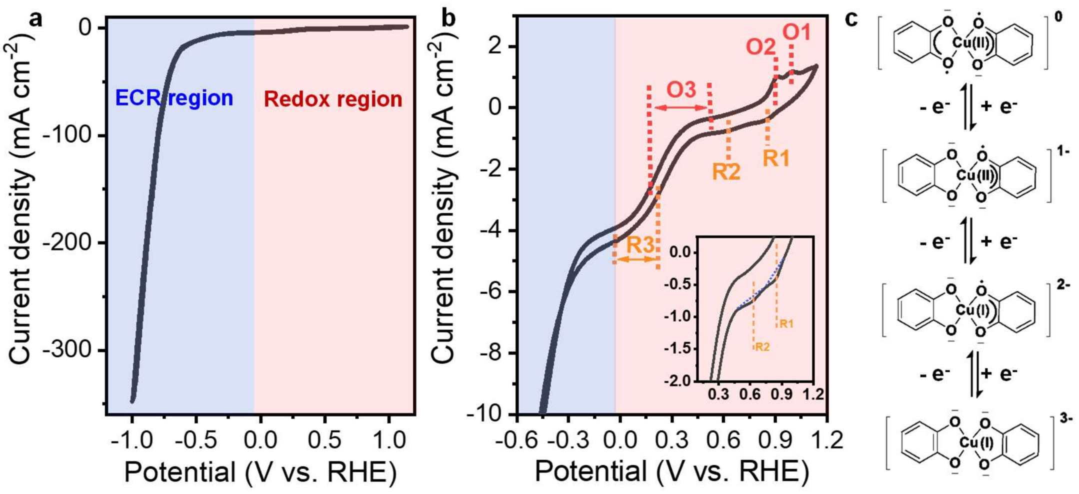

Supplementary Fig. 31 Redox properties analysis of Cu-DBC. a CV curve of Cu-DBC with the voltage window containing both ECR and redox of the Cu-DBC. **b** The enlarged display of redox region (red region) that revealing the instinct redox property of the Cu-DBC. The inset is the enlarged display of R1 and R2. **c** The redox states of Cu-DBC[1]. R1~R3 represent the reduction peaks and O1~O3 represent the oxidation peaks.

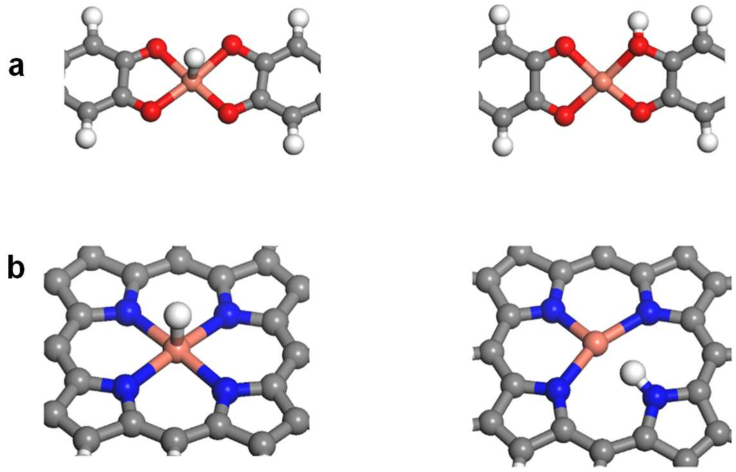

Supplementary Fig. 32 Modeled structure (left) and optimized structure (right) for *H adsorption on catalytic sites. a Cu-O_4 and **b** porphyrin Cu-N_4 sites. Grey, blue, red, white, and orange spheres represent C, N, O, H, and Cu atoms, respectively.

Supplementary Table 5 Löwdin charge analysis of the Cu-O_4 and Cu-N_4 systems. The results are obtained from Löwdin population analysis by ORCA calculations.

**Model**	**Löwdin Charge**
Cu-O_4	0.39
Cu-O_4H_1	0.28
Cu-O_4H_4	0.04
Porphyrin Cu-N_4	0.18
Porphyrin Cu-N_4H_1	0.12
Phthalocyanine Cu-N_4	0.19

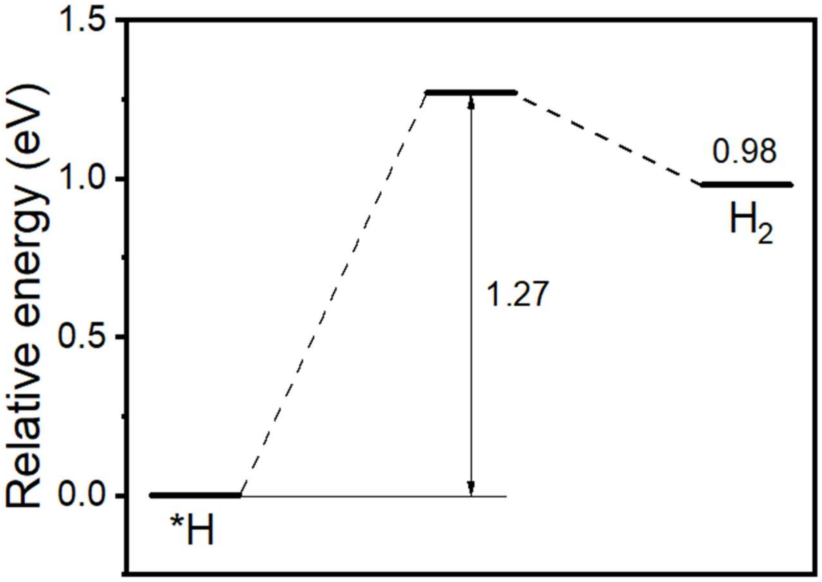

Supplementary Fig. 33 The energy barrier of the formation of H_2 (i.e. full HER process) for Cu-O_4 system following the Volmer-Tafel mechanism.

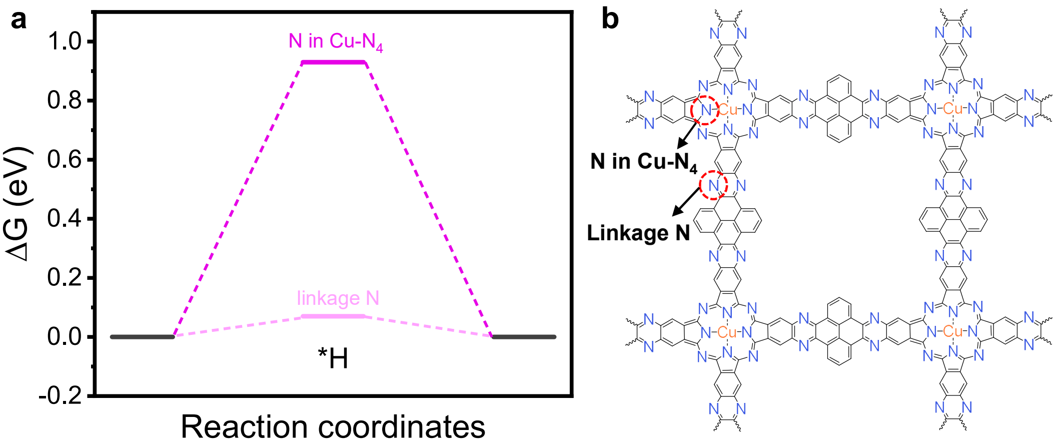

Supplementary Fig. 34 The ananlysis of electroreduction active sites in Cu-PPCOF. **a** The energy profiles of *H adsorption on different N sites in CuPPCOF. **b** structural scheme of the related N sites in CuPPCOF.

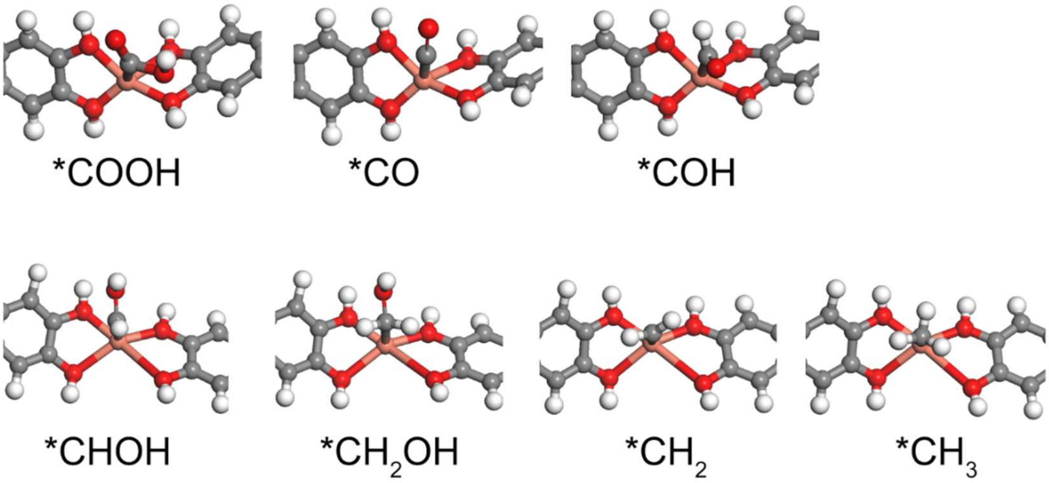

Supplementary Fig. 35 Structures of the reaction intermediates involved in the proposed reaction mechanism for the ECR-to-CH_4 on Cu-O_4 site in Cu-DBC catalyst.

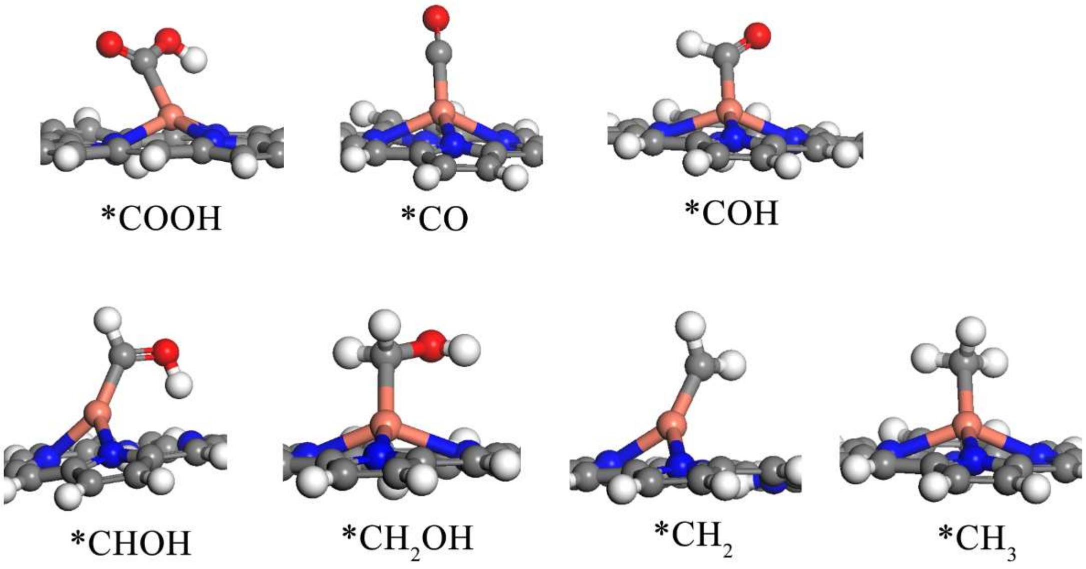

Supplementary Fig. 36 Structures of the reaction intermediates involved in the proposed reaction mechanism for the ECR-to-CH_4 on Cu-N_4 site in Cu-TTCOF catalyst.

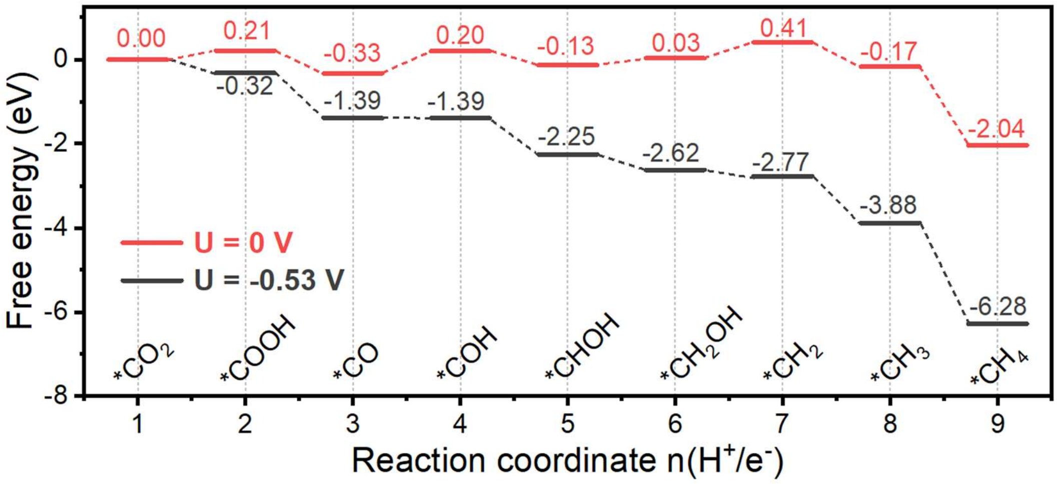

Supplementary Fig. 37 The energy profiles of Cu-O_4 sites in Cu-DBC with U = 0 and U = U_{Onset}.

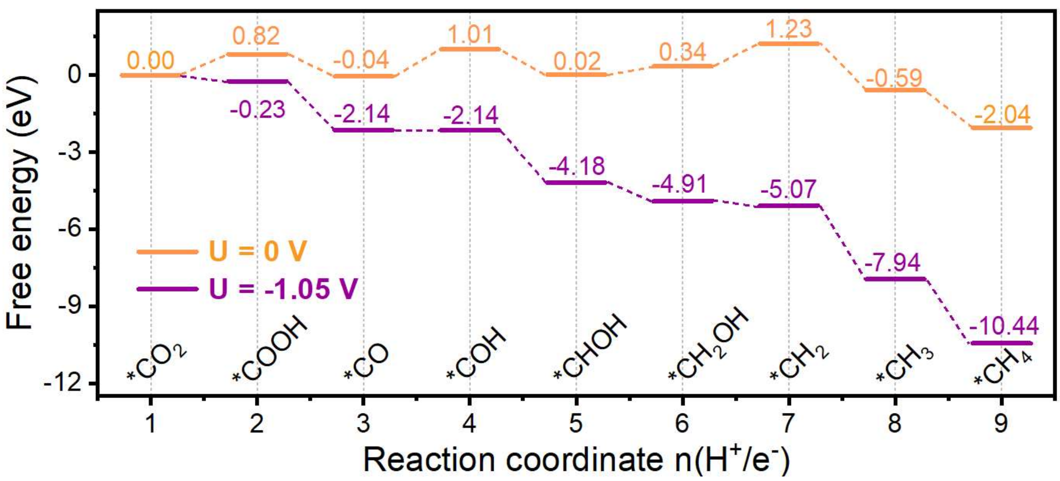

Supplementary Fig. 38 The energy profiles of porphyrin Cu-N_4 in Cu-TTCOF with U = 0 and U = U_{Onset}.

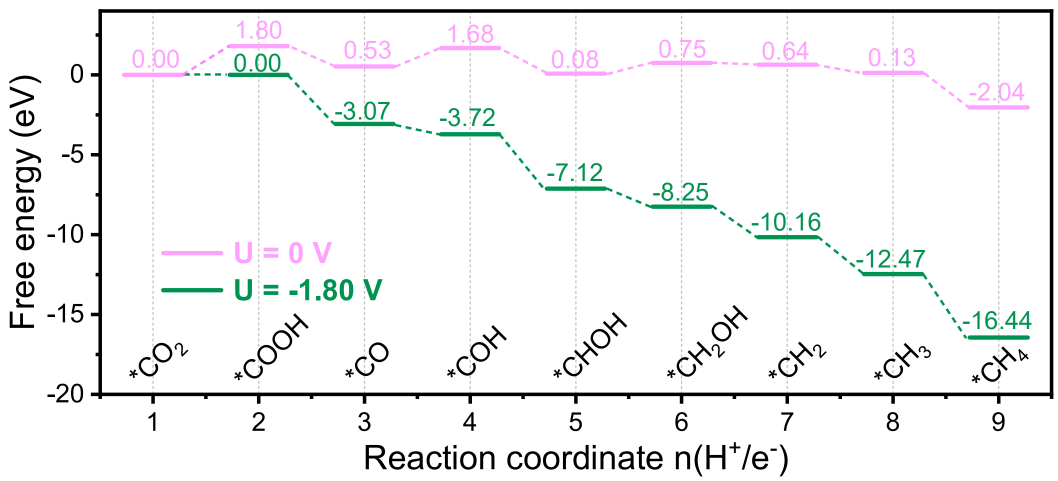

Supplementary Fig. 39 The energy profiles of phthalocyanine Cu-N_4 in Cu-PPCOF with U = 0 and U = U_{Onset}.

Supplementary Table 6 Electrochemical performance of CO_2 reduction to CH_4 of Cu-based electrocatalysts

Catalyst	FE	*j* (mA cm^2)	Potential (V vs. RHE)	Cell Type	Ref.
Cu-P-ED	85%	40	-2.8 vs. SCE	H-Type	11
Cu clusters/DRC	81.7%	25	-1.0	H-Type	12
N-Cu/C	80%	12	-1.35	H-Type	13
Cu_2O@CuHHTP	73%	18	-1.4	H-Type	14
CuS NSs	73%	8	-1.1	H-Type	15
CuPc	66%	18	-1.05	H-Type	16
CuO@Cu-MOF	63.2%	14.5	-1.7	H-Type	17
HATNA-Cu-MOF	78%	10.5	-1.5	H-Type	18
Cu/La_2CuO_4	53.6%	215	-1.4	Flow cell	19
Cu_{oh}	53%	100	-0.91	Flow cell	20
NNU-33(H)	82%	391	-0.9	Flow cell	21
Cu-DBC	80%	206	-0.9	Flow cell	This work

The structure models (with coordinates) of DFT calculations.

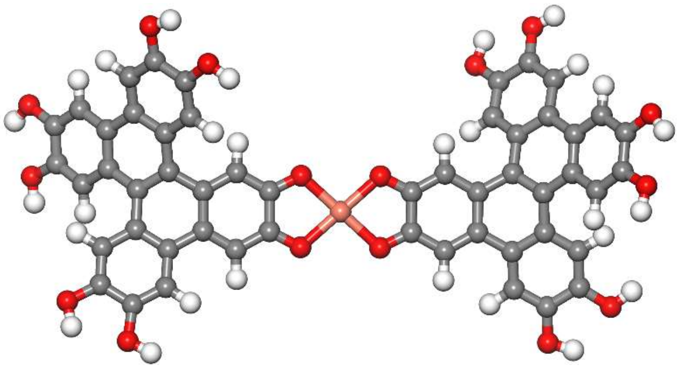

97

Coordinates of the Cu-O_4 site in Cu-DBC system

C	8.57947500	1.87379400	0.37645800
C	5.03027400	-0.30535400	-0.50678300
C	3.81683000	0.37771300	-0.39011500
C	2.59811100	-0.30480700	-0.27734600
C	2.59666900	-1.77077100	-0.27397300
C	3.82506400	-2.45204900	-0.34343700
C	6.30175900	-2.46614600	-0.51091800
C	7.49413100	-1.73892900	-0.28139700
C	8.72369000	-2.41357000	-0.43978400
C	8.51660700	0.46142400	0.53651500
C	8.77193000	-3.75661800	-0.75562200
C	7.58615100	-4.49029000	-0.93131100
C	6.37542200	-3.83655500	-0.82089500
C	7.60450000	2.52331600	-0.47869100
C	6.44678900	1.80455800	-0.88181900
C	5.54259700	2.43268800	-1.77003300
C	5.73196900	3.72565200	-2.20442500
C	6.85875800	4.44724200	-1.75959100
C	9.43056700	-0.14874500	1.43558600
C	7.77577400	3.85118700	-0.92676800
C	6.30297900	0.40213500	-0.50891900
C	7.44089400	-0.28947600	-0.07818600
C	-7.59807100	2.53876300	0.48194200
C	-6.43557900	1.81314300	0.85626800
C	-5.50668300	2.44903200	1.71639700
C	-5.68782900	3.74579900	2.13498900
C	-6.81938300	4.48010500	1.72454500
C	10.38293200	0.58637500	2.09812300
C	-7.75556200	3.86948100	0.92153400
C	-6.30600900	-2.45597300	0.50277800
C	-7.50533200	-1.73308400	0.29108800
C	-8.73168100	-2.40725100	0.45876400

C	-8.78683500	-3.75585800	0.75678500
C	-7.59074900	-4.47645300	0.91843900
C	-6.37928900	-3.83012800	0.80836800
C	10.47932300	1.97955900	1.89504300
C	-5.03064900	-1.75909100	0.42780300
C	-5.03595100	-0.30028700	0.47063900
C	-3.82164000	0.37949800	0.33122100
C	-2.60354000	-0.30360000	0.23010800
C	-2.60198600	-1.76679000	0.24050600
C	-3.82863200	-2.44618400	0.32131500
C	-8.59685000	1.88551800	-0.34530800
C	9.57955400	2.59815900	1.05603100
C	-8.54412800	0.47149600	-0.50167500
C	-9.49045800	-0.13820200	-1.36702900
C	-10.46359200	0.59791200	-1.99587100
C	-10.54428000	1.99352900	-1.79948900
C	-9.61378600	2.61261800	-0.99486900
C	-7.45531500	-0.28148500	0.08526800
C	-6.30662700	0.40893200	0.49184400
C	5.02468400	-1.76465600	-0.44897700
Cu	-0.00309200	-1.03580700	-0.02312800
O	1.45217600	0.27190900	-0.14953500
O	1.45469900	-2.35077900	-0.16977100
O	9.93038500	-4.47237200	-0.94682200
O	7.62891100	-5.81162800	-1.24559000
O	4.85702300	4.29122800	-3.07678800
O	6.95688100	5.72489000	-2.25555100
O	-4.84337000	4.42037400	2.98406000
O	-6.99576900	5.76031500	2.14466500
O	-9.98849700	-4.36952400	0.91857600
O	-7.75274600	-5.80689700	1.22293000
O	-1.45508300	0.27298800	0.10563900
O	-1.45838100	-2.34788400	0.13557300
O	-11.39725700	0.08878300	-2.86731600
O	-11.50621500	2.71722800	-2.42594100
O	11.28179000	0.07706500	3.00764000
O	11.42107500	2.70433200	2.55255300
H	4.69972800	1.89501300	-2.17989800
H	9.65415600	-1.86050900	-0.38072400
H	3.79242100	-3.53171800	-0.27151700
H	-4.66311400	1.89235100	2.10556300
H	-8.63620100	4.44448400	0.66656700
H	8.66304900	4.40962100	-0.64665200
H	9.33296800	-1.20356500	1.66498400

H	-9.67126800	-1.87452600	0.42010000
H	-5.47098200	-4.39241300	0.99750600
H	-9.67504300	3.68966500	-0.91028400
H	-3.78901900	1.45518700	0.23609500
H	9.64518600	3.67577200	0.97730600
H	-9.40985000	-1.19431700	-1.59412100
H	-3.79314800	-3.52616500	0.25207800
H	5.48286300	-4.41369000	-1.02447000
H	3.78169200	1.45543400	-0.32335700
H	10.69671500	-3.88952300	-0.92155800
H	8.55489300	-6.07586000	-1.32903600
H	5.15982200	5.18723900	-3.27571400
H	7.75476700	6.15477700	-1.92986800
H	-4.06242300	3.89029200	3.17673800
H	-6.24528500	6.00135100	2.70432800
H	-9.82851400	-5.29215200	1.15946800
H	-6.89897900	-6.22986900	1.36375000
H	-11.29746300	-0.86554500	-2.95107400
H	-12.04063100	2.11480800	-2.96104100
H	11.09047800	-0.84903800	3.19007400
H	11.91593200	2.10740800	3.13011400

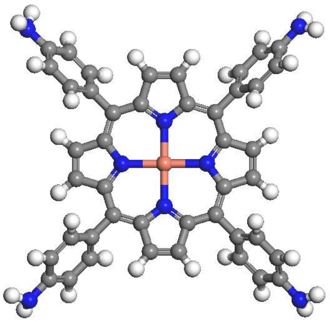

85

Coordinates of porphyrin Cu-N_4 site in Cu-TTCOF

C	35.80955538989398	21.73451790495152	5.72033529391110
C	27.45552898304795	21.39130324464108	4.00813823949699
C	27.64333991385484	22.73329241665391	3.90265158051780
C	35.62703728423263	20.38982846683460	5.80112241991481
C	34.28041551131461	23.70647767358526	5.45738388836746
C	28.88188338954406	19.46635441785877	4.75680899709808
C	29.55421825043714	24.28651340755320	4.38943334174255
C	33.62060692203413	18.88082364484237	5.76560009023845
C	34.50473849709700	22.33075147672445	5.56510334044910
C	28.68912049945070	20.82778026181739	4.50146858951348
C	28.99349842807403	23.00701401620000	4.33216111436477

C	34.20796201881031	20.14800256538026	5.70003923463260
C	31.62165711639371	17.36326925460556	5.75862148271348
C	31.45343197450208	25.85273985548044	4.88130953522536
C	30.30103087761285	17.52710777415999	5.48309339438020
C	32.76941587158814	25.69136912046309	5.18072684044184
C	32.25343364929960	18.64916550314230	5.58606939476500
C	30.88987137040029	24.53375077443806	4.72102259279819
C	30.10965544648011	18.91434889308436	5.13478443861326
C	33.02681135678316	24.27183634541884	5.20591602274316
H	36.73985265429733	22.28083815993307	5.74106810535971
H	26.57438476796555	20.82346291655542	3.75349598122496
H	26.94513271916390	23.47453826461470	3.54727496209437
H	36.37938118171336	19.62334186021702	5.90137253525542
H	32.12567269927914	16.46349363218654	6.07413242709863
H	30.90167571188540	26.77591258834824	4.79702472732132
H	29.51764716114075	16.78702036143491	5.53003549945335
H	33.50126760560902	26.45619963240943	5.38795013701734
N	33.54659641973191	21.34358520966034	5.53711606983828
N	29.61788555169740	21.82855851894742	4.67151108471575
N	31.30878756084683	19.58305852592110	5.22609493867104
N	31.86298481639406	23.58653551723206	4.94269282449789
Cu	31.58400089077691	21.58547484974139	5.09473613626719
C	28.67887589252928	25.45120672999030	4.08124576378614
C	28.93105182425777	26.27629542068046	2.97919246660071
C	27.57450440789890	25.76025939278526	4.88385511432951
C	28.11791647574785	27.36316237333877	2.68741898156926
H	29.77767587573760	26.05575111400388	2.33778880202326
C	26.75683791160183	26.84683665636429	4.60426871437623
H	27.36176975271595	25.14104770418503	5.74901293369707
C	27.01508068815045	27.66993990603692	3.49812246495995
H	28.32898303939610	27.97764527435826	1.81621805391292
H	25.90858230867256	27.06308105401085	5.24805097500475
C	35.44425639762654	24.62275793170554	5.61552267556669
C	35.88930289827162	25.41810057232072	4.55325429911091
C	36.13179049940889	24.71815266557698	6.83080033875114
C	36.97577122317853	26.27124769818348	4.69287156205583
H	35.37858750744606	25.35602944302814	3.59805567746203
C	37.21622203982528	25.57162090124022	6.98366405080252
H	35.80262746305201	24.11720257009904	7.67183306445561
C	37.66021379186606	26.36325816400347	5.91386685324081
H	37.30101391541110	26.87333533590991	3.84881946381184
H	37.72069759317505	25.63678056850365	7.94406025668047
C	34.50061028090863	17.71075980728611	6.03895332561942
C	34.66693515222855	16.69524712733375	5.08991216453395

C	35.19234447576442	17.58733153481402	7.24945540730986
C	35.49160337952450	15.60516055872930	5.33298731153507
H	34.14842382841066	16.77038280579707	4.14001387596000
C	36.01617369575891	16.49935632473336	7.50540308843392
H	35.07447466493621	18.35673731084168	8.00550102078611
C	36.18345804742412	15.48824685961932	6.54746049803531
H	35.60512054207564	14.83418915887992	4.57516958910019
H	36.52750759267127	16.42281307125080	8.46123778739477
C	27.71487761944388	18.55266100044253	4.61292342852124
C	27.70367180614001	17.53694970248066	3.64987807990066
C	26.58883817932424	18.67993873926540	5.43427203431704
C	26.61623977531312	16.68587381048669	3.50725882269025
H	28.56152152614800	17.42093958583570	2.99558945737952
C	25.49749496934685	17.83167056169378	5.30371431977114
H	26.57415619991419	19.45585924888637	6.19265675792397
C	25.49034907631886	16.81938533691821	4.33302911151624
H	26.63612547793399	15.90728092683043	2.74894618664606
H	24.64286445805820	17.94561465914042	5.96570561575176
N	24.42022433336820	15.93014682155592	4.23205675730475
H	23.54241630283941	16.27493401393594	4.59175757231522
H	24.30801666472943	15.49777088314520	3.32687500502393
N	26.16219563268026	28.72664484020229	3.17709331306733
H	25.64172597674150	29.10610589637625	3.95431588951678
H	26.57744461858007	29.45566309028878	2.61568514302039
N	38.71357807790476	27.26350913743188	6.07858858932897
H	39.36307617642753	27.03042611481232	6.81537551830340
H	39.19113611998102	27.52336317386973	5.22812335291994
N	36.96697920062097	14.36630036052880	6.81977281999204
H	37.31449672089824	13.88071402666787	6.00577908022416
H	37.69262477026255	14.50818746855754	7.50709698291613

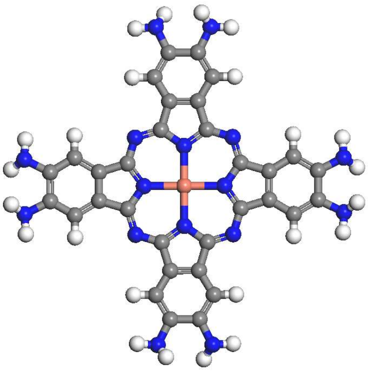

73

Coordinates of phthalocyanine Cu-N_4 sites in Cu-PPCOF

C	34.59981224372083	22.10996991282352	4.36015782537410
C	28.78751654705603	20.80482277078269	3.99323548867431

C	29.18021128426527	22.98234040317173	3.66290344730710
C	34.20856398623931	19.93003816814835	4.67622521618360
C	32.30543168287644	18.58445606886867	4.70388056744421
C	31.08281957398950	24.32876121041815	3.64062030243659
C	30.11373843610851	18.93779992896948	4.42888844532381
C	33.27279839814591	23.97834927648104	3.93267854432759
N	33.62186952155285	21.14607686459331	4.42016779543709
N	29.76609671023779	21.76767845051578	3.92710473449734
N	31.34883332449357	19.53932407673886	4.45424129343349
N	32.03906401168225	23.37448113511826	3.89369362279546
Cu	31.69444428277848	21.45708458322087	4.17329247979969
N	29.77529879307381	24.15678811367007	3.52843929209650
N	34.44805620915132	23.40657762586149	4.14153012375395
N	33.61369954993061	18.75511697692061	4.80794658789834
N	28.93814449843568	19.51004227570379	4.22267445435001
C	33.10705797524345	25.40436489878093	3.69767321374492
C	31.73298540769634	25.62347005923197	3.50986282784523
C	34.01207172309643	26.46248789486767	3.66093565048370
C	31.24002314315448	26.89885441377955	3.24468897208884
C	33.52808161648476	27.74403328538824	3.42069786021771
H	35.06947337268295	26.29039580905670	3.83752937238156
C	32.13753240444376	27.95931312444167	3.18292209057442
H	30.17927184774916	27.05538021757409	3.07447123577271
C	35.64715848198151	20.11551998970629	4.78849580665497
C	35.89305263147260	21.48319574183421	4.58584280662906
C	36.69041974718031	19.23226852377768	5.05607878574546
C	37.19050627494613	21.98925202304341	4.60816557403839
C	37.98789943998619	19.73083716587688	5.10468554870268
H	36.48919753954539	18.18190119493742	5.24274427336465
C	38.24220040870033	21.11219128309893	4.85078123173217
H	37.37459256030225	23.04258972056795	4.42018831537635
C	27.74162898960884	22.79672186254065	3.54960160122142
C	27.49486616604270	21.43064964106978	3.76144575809468
C	26.69900978834989	23.67890887725154	3.27632195747882
C	26.19714762700577	20.92510516421420	3.74095907314229
C	25.40124918688554	23.18069205321922	3.22965507775209
H	26.90076348635887	24.72815973402889	3.08410444803020
C	25.14619899678420	21.80097680223174	3.49125003694644
H	26.01239929568529	19.87296179058352	3.93482998006053
C	30.27824362673240	17.51340937215540	4.67336853094587
C	31.65377023982118	17.29145043967133	4.84670583589091
C	29.36700717466789	16.46406669743023	4.76632562031915
C	32.14950569137719	16.01183743950956	5.08472970296731
C	29.84858630444360	15.18513402520225	5.02373146238118

H	28.30224101198150	16.64694859781475	4.65655866151396
C	31.25026963636730	14.95311435469167	5.15933287293922
H	33.21717075425659	15.84516269612763	5.19112525355487
N	34.36490429193991	28.87083163670413	3.33928409526169
N	31.73384321383290	29.28594007893740	2.95366598811742
N	39.10890106446077	18.91587882641969	5.33992942345193
N	39.58180682158309	21.53112392741276	4.92319774300038
N	31.64863394134737	13.63340758979250	5.42836901703588
N	29.01195859354705	14.05721327755594	5.10413468364970
N	23.80629509654616	21.38235025389617	3.41914287785508
N	24.28102585978190	23.99484478528339	2.98884010627640
H	40.21042136625405	20.90679180979632	4.42931567369943
H	39.71895628992996	22.48536964385940	4.61895204279523
H	39.76165730067719	19.33596532609186	5.99339877298376
H	38.86793912406628	17.97895149919656	5.63278264888310
H	32.64989574649004	13.50020390904930	5.38747401932806
H	31.17284689875888	12.95711751199004	4.84097391352064
H	29.23489391684539	13.46615191598696	5.89855816747532
H	28.02821550309561	14.29037686467439	5.10304967795823
H	23.66878290656037	20.42862569597122	3.72476878783231
H	23.17769861551934	22.00750102345322	3.91206205030344
H	24.52214338619872	24.92997835315898	2.69059972595890
H	23.62769189171411	23.57071947442748	2.33865312959787
H	32.33757287369134	29.76692525506582	2.29564789133807
H	30.76661525363166	29.36590478403219	2.67092326687563
H	35.31343297575465	28.68527500109682	3.63530422908306
H	33.98779894300026	29.66923041043457	3.83863610799342

Supplementary References

1. Liu, J., Zhou, Y., Xie, Z., Li, Y., Liu, Y., Sun, J., Ma, Y., Terasaki, O., Chen, L. Conjugated copper-catecholate framework electrodes for efficient energy storage. *Angew. Chem. Int. Ed.* **59**, 1081-1086 (2020).
2. Kovtyukhova, N. I., Ollivier, P. J., Martin, B. R., Mallouk, T. E., Chizhik, S. A., Buzaneva, E. V., Gorchinskiy, A. D. Layer-by-layer assembly of ultrathin composite films from micron-sized graphite oxide sheets and polycations. *Chem. Mater.* **11**, 771-778 (1999).
3. Lu, M., Liu, J., Li, Q., Zhang, M., Liu, M., Wang, J.-L., Yuan, D.-Q., Lan, Y.-Q. Rational design of crystalline covalent organic frameworks for efficient CO_2 photoreduction with H_2O. *Angew. Chem. Int. Ed.* **58**, 12392-12397 (2019).
4. Huang, N., Lee, K. H., Yue, Y., Xu, X., Irle, S., Jiang, Q., Jiang, D. A stable and conductive metallophthalocyanine framework for electrocatalytic carbon dioxide reduction in water. *Angew. Chem. Int. Ed.* **59**, 16587-16593 (2020).
5. Neese, F. The ORCA program system. *WIRES Comput. Mol. Sci.* **2**, 73-78 (2012).
6. Schäfer, A., Huber, C., Ahlrichs, R. Fully optimized contracted Gaussian basis sets of triple zeta valence quality for atoms Li to Kr. *J. Chem. Phys.* **100**, 5829-5835 (1994).
7. Weigend, F., Häser, M., Patzelt, H., Ahlrichs, R. RI-MP2: optimized auxiliary basis sets and demonstration of efficiency. *Chem. Phys. Lett.* **294**, 143-152 (1998).
8. Weigend, F. Accurate Coulomb-fitting basis sets for H to Rn. *Phys. Chem. Chem. Phys.* **8**, 1057-1065 (2006).
9. Grimme, S., Antony, J., Ehrlich, S., Krieg, H. A consistent and accurate ab initio parametrization of density functional dispersion correction (DFT-D) for the 94 elements H-Pu. *J. Chem. Phys.* **132**, 154104 (2010).
10. Peterson, A. A., Abild-Pedersen, F., Studt, F., Rossmeisl, J., Nørskov, J. K. How copper catalyzes the electroreduction of carbon dioxide into hydrocarbon fuels. *Energy Environ. Sci.* **3**, 1311-1315 (2010).
11. Qiu, Y.-L., Zhong, H.-X., Zhang, T.-T., Xu, W.-B., Li, X.-F., Zhang, H.-M. Copper electrode fabricated via pulse electrodeposition: toward high methane selectivity and activity for CO_2 electroreduction. *ACS Catal.* **7**, 6302-6310 (2017).
12. Hu, Q., Han, Z., Wang, X., Li, G., Wang, Z., Huang, X., Yang, H., Ren, X., Zhang, Q., Liu, J., He, C. Facile synthesis of sub-nanometric copper clusters by double confinement enables selective reduction of carbon dioxide to methane. *Angew. Chem. Int. Ed.* **59**, 19054-19059 (2020).
13. Manthiram, K., Beberwyck, B. J., Aivisatos, A. P. Enhanced electrochemical methanation of carbon dioxide with a dispersible nanoscale copper catalyst. *J. Am. Chem. Soc.* **136**, 13319-13325 (2014).
14. Yi, J.-D., Xie, R., Xie, Z.-L., Chai, G.-L., Liu, T.-F., Chen, R.-P., Huang, Y.-B., Cao, R. Highly selective CO_2 electroreduction to CH_4 by in-situ generated Cu_2O single-type sites on a conductive MOF: stabilizing key intermediates with hydrogen bonding. *Angew. Chem. Int. Ed.* **59**, 23641-23648 (2020).
15. Zhao, Z., Peng, X., Liu, X., Sun, X., Shi, J., Han, L., Li, G., Luo, J. Efficient and stable electroreduction of CO_2 to CH_4 on CuS nanosheet arrays. *J. Mater. Chem.*

A **5**, 20239-20243 (2017).

16. Weng, Z., Wu, Y., Wang, M., Jiang, J., Yang, K., Huo, S., Wang, X.-F., Ma, Q., Brudvig, G. W., Batista, V. S., Liang, Y., Feng, Z., Wang, H. Active sites of copper-complex catalytic materials for electrochemical carbon dioxide reduction. *Nat. Commun.* **9**, 415 (2018).
17. Tan, X., Yu, C., Zhao, C., Huang, H., Yao, X., Han, X., Guo, W., Cui, S., Huang, H., Qiu, J. Restructuring of Cu_2O to Cu_2O@Cu metal organic frameworks for selective electrochemical reduction of CO_2. *ACS Appl. Mater. Interfaces* **11**, 9904-9910 (2019).
18. Liu, Y.; Li, S.; Dai, L.; Li, J.; Lv, J.; Zhu, Z.; Yin, A.; Li, P.-F.; Wang, B. The synthesis of hexaazatrinaphthylene based 2D conjugated copper metal-organic framework for highly selective and stable electroreduction of CO_2 to methane. *Angew. Chem. Int. Ed.* **60**, 14473-14479 (2021).
19. Chen, S., Su, Y., Deng, P., Qi, R., Zhu, J., Chen, J., Wang, Z., Zhou, L., Guo, X., Xia, B. Y. Highly selective carbon dioxide electroreduction on structure-evolved copper perovskite oxide toward methane production. *ACS Catal.* **10**, 4640-4646 (2020).
20. De Gregorio, G. L., Burdyny, T., Loiudice, A., Iyengar, P., Smith, W. A., Buonsanti, R. Facet-dependent selectivity of Cu catalysts in electrochemical CO_2 reduction at commercially viable current densities. *ACS Catal.* **10**, 4854-4862 (2020).
21. Zhang, L., Li, X.-X., Lang, Z.-L., Liu, Y., Liu, J., Yuan, L., Lu, W.-Y., Xia, Y.-S., Dong, L.-Z., Yuan, D.-Q., Lan, Y.-Q. Enhanced cuprophilic interactions in crystalline catalysts facilitate the highly selective electroreduction of CO_2 to CH_4. *J. Am. Chem. Soc.* **143**, 3808-3816 (2021).
