# Supplementary material for: Coordination environment dependent selectivity of single-site-Cu enriched crystalline porous catalysts in CO2 reduction to CH4
Source: Nat Commun. 2021 Nov 4;12:6390. doi: 10.1038/s41467-021-26724-8 (PMC8568903; doi:10.1038/s41467-021-26724-8)
Supplement: Supplementary file 3 — Description of Additional Supplementary Files [file 41467_2021_26724_MOESM3_ESM.pdf]

Description of Additional Supplementary Files

File Name: Supplementary Movie 1
Description: The stretching vibration of Peak 1 of the simulated Raman spectrum shown in Supplementary Fig. 15b.

File Name: Supplementary Movie 2
Description: The stretching vibration of Peak 2 of the simulated Raman spectrum shown in Supplementary Fig. 15b.

File Name: Supplementary Movie 3
Description: The stretching vibration of Peak 3 of the simulated Raman spectrum shown in Supplementary Fig. 15b.
