# Supplementary figures and images for: Coordination environment dependent selectivity of single-site-Cu enriched crystalline porous catalysts in CO2 reduction to CH4
Source: Nat Commun. 2021 Nov 4;12:6390. doi: 10.1038/s41467-021-26724-8 (PMC8568903; doi:10.1038/s41467-021-26724-8)

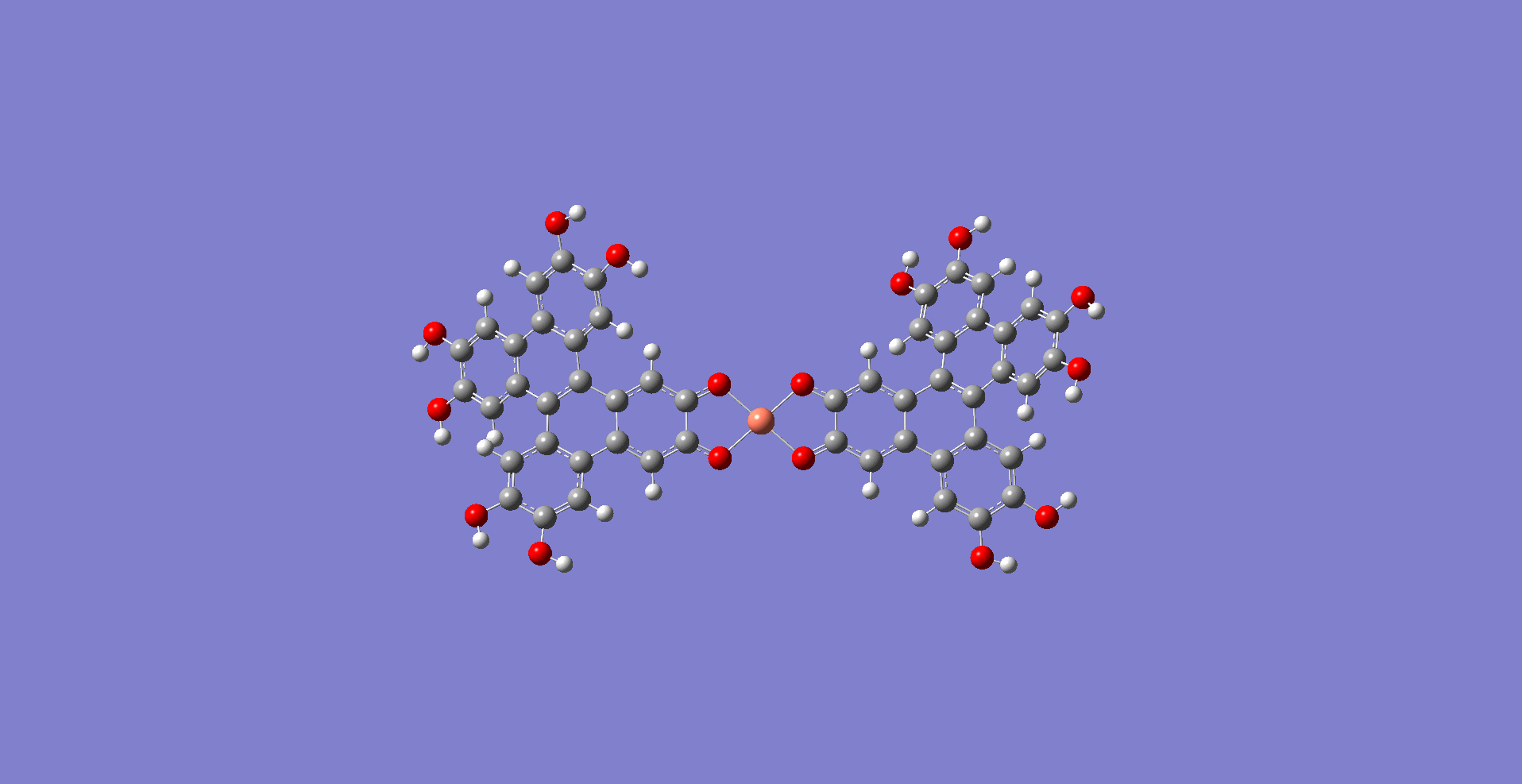

Supplement: Supplementary file 4 — Supplementary Movie 1 [file 41467_2021_26724_MOESM4_ESM.gif]

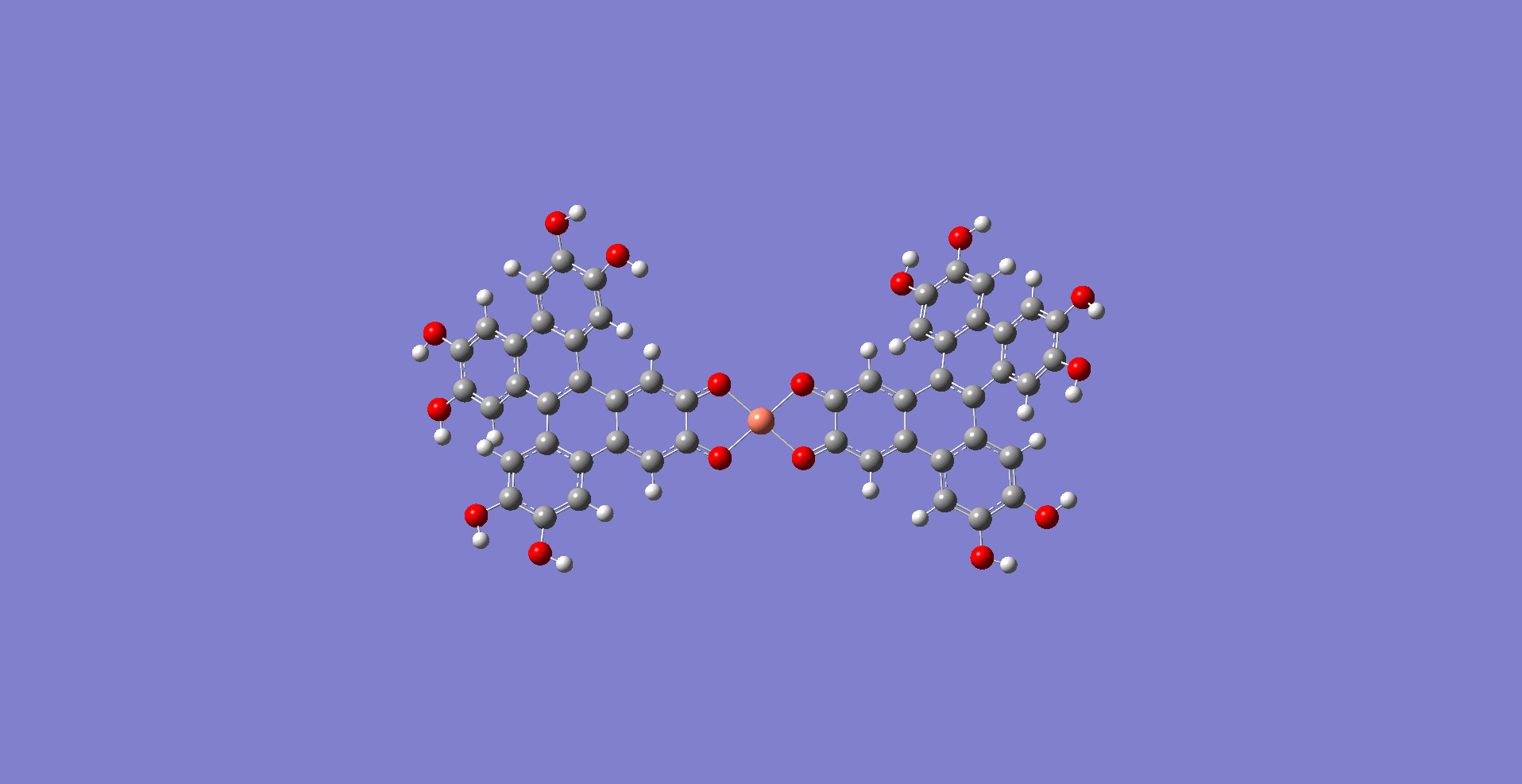

Supplement: Supplementary file 5 — Supplementary Movie 2 [file 41467_2021_26724_MOESM5_ESM.gif]

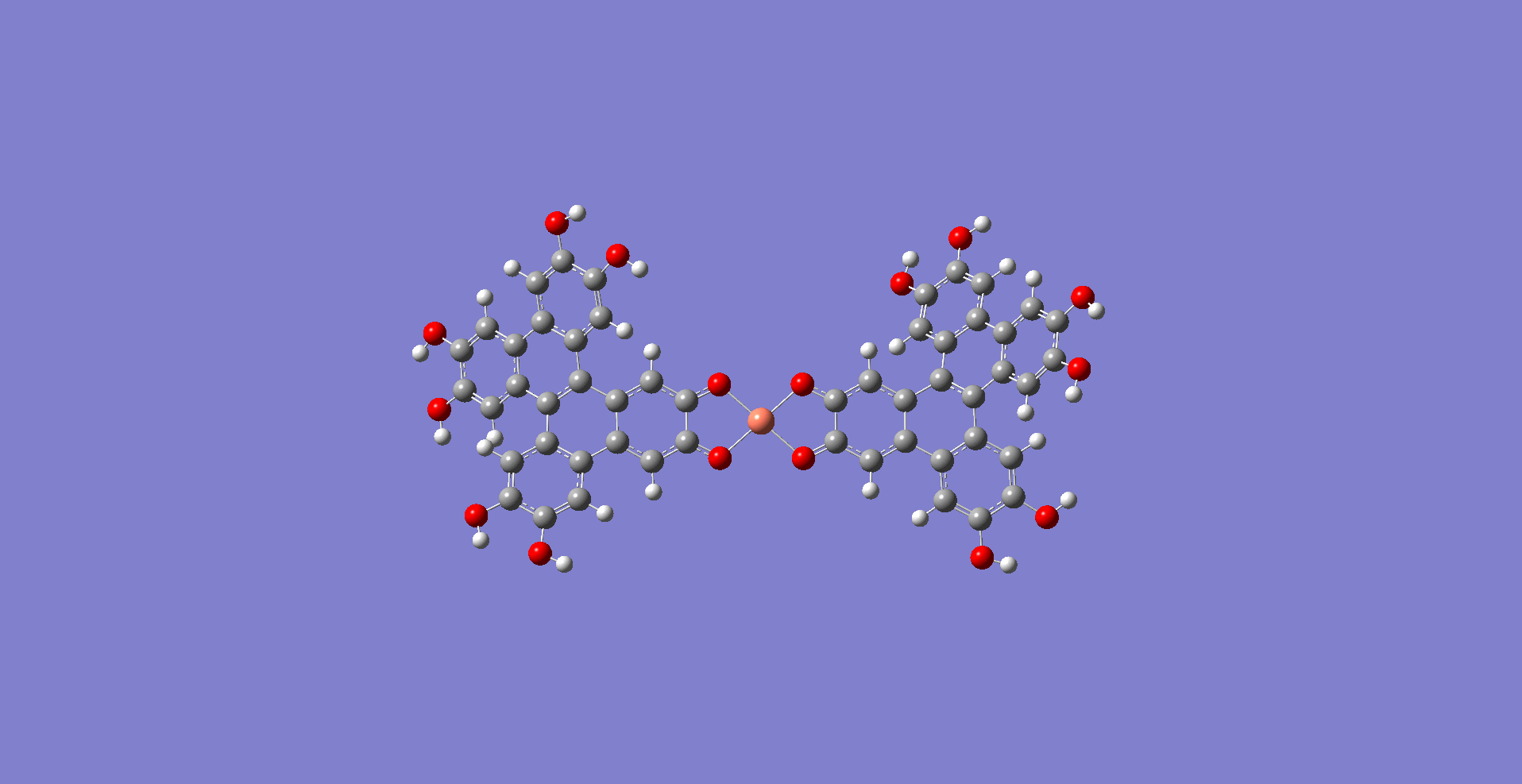

Supplement: Supplementary file 6 — Supplementary Movie 3 [file 41467_2021_26724_MOESM6_ESM.gif]
